# International society of sports nutrition position stand: nutrition and weight cut strategies for mixed martial arts and other combat sports

**DOI:** 10.1080/15502783.2025.2467909

**Published:** 2025-03-09

**Authors:** Anthony A. Ricci, Cassandra Evans, Charles Stull, Corey A. Peacock, Duncan N. French, Jeffery R. Stout, David H. Fukuda, Paul La Bounty, Douglas Kalman, Andrew J. Galpin, Jaime Tartar, Sarah Johnson, Richard B. Kreider, Chad M. Kerksick, Bill I. Campbell, Aaron Jeffery, Chris Algieri, Jose Antonio

**Affiliations:** a Nova Southeastern University, Department of Health and Human Performance, Fight Science Lab, Fort Lauderdale, FL, USA; b Nova Southeastern University, Department of Psychology and Neuroscience, Fort Lauderdale, FL, USA; c UFC Performance Institute, Las Vegas, NV, USA; d University of Central Florida, Institute of Exercise Physiology and Rehabilitation Science, School of Kinesiology and Rehabilitation Sciences, Orlando, FL, USA; e University of Mary Hardin Baylor, Mayborn College of Health Sciences, Belton, TX, USA; f Nova Southeastern University, Department of Nutrition, Fort Lauderdale, FL, USA; g Parker University, Human Performance Center, Dallas, TX, USA; h Texas A&M University, Exercise & Sport Nutrition Lab, Department of Kinesiology and Sports Management, College Station, TX, USA; i Lindenwood University, Exercise and Performance Nutrition Laboratory, St. Charles, MO, USA; j University of South Florida, Performance & Physique Enhancement Laboratory, Tampa, FL, USA; k Kill Cliff FC, Deerfield Beach, FL, USA; l Nova Southeastern University, Department of Psychology and Neuroscience, Davie, FL USA

**Keywords:** Longitudinal weight descent, acute weight loss, acute water loss, nutrition, performance

## Abstract

Following an extensive literature review, the International Society of Sports Nutrition (ISSN) has developed an official position on nutritional and weight cut strategies for combat sports. The type of combat sport, length of the fight camp, and time between weigh-in and competition are factors influencing nutritional and weight cut strategies. The following 16 points constitute the Position Statement of the Society; the Research Committee has approved them. 1. Combat sports have differing weight categories, official weigh-in times, and competition frequencies, influencing the nutritional and weight cut strategies for training and competition. 2. As the duration of a combat match increases, >4 min, contribution of the aerobic system can rise to >70%, yet anaerobic alactic pathways and anaerobic glycolytic pathways support high-output bursts. 3. During the off camp/general preparation phase, athletes should maintain a weight ranging 12% to 15% above the weight division requirement. 4. Supplements including creatine, beta-alanine, beta-hydroxy-beta-methylbutyrate, and caffeine have been shown to enhance performance and/or recovery during preparation phases, competition, and post-competition. 5. During fight camp, strategic decreases in calorie intake are necessary for an efficient longitudinal weight descent. Individual caloric needs can be determined using indirect calorimetry or validated equations such as Mifflin St. Jeor or Cunningham. 6. Protein should be prioritized during longitudinal weight descents to preserve lean body mass, and the timely delivery of carbohydrates supports training demands. Macronutrients should not drop below the following: carbohydrates 3.0–4.0 g/kg, protein 1.2–2.0 g/kg, and fat 0.5 to 1.0 g/kg/day. 7. Suitable losses in body mass range from 6.7% at 72 h, 5.7% at 48 h, and 4.4% at 24 h, prior to weigh-in. 8. Sodium restriction and water loading are effective for inducing polyuria and acute water loss. 9. During fight week, water-bound glycogen stores can be depleted through exercise and carbohydrate restriction, facilitating a 1% to 2% loss in body mass, with equivalent losses from a low-fiber intake of <10 g/day for 4 days. 10. During fight week, acute water loss strategies, including sauna, hot water immersion, and mummy wraps, can be used effectively with appropriate supervision (optimally ~2–4% of body mass within 24 h of weigh-in). 11. Post-weigh-in, rapid weight gain strategies are utilized to recover lost body fluid/mass before competition with the intent of gaining a competitive advantage. 12. Oral rehydration solutions (1 to 1.5 liters/h) combined with a sodium range of 50–90 mmol/dL should take precedence immediately post-weigh-in. 13. Fast-acting carbohydrates at a tolerable rate of ≤ 60 g/h should follow oral rehydration solutions. Post weigh-in intake of fiber should be limited to avoid gastrointestinal distress. 14. Post-weigh-in carbohydrate intake at 8–12 g/kg may be appropriate for combat athletes that undertook significant glycogen depletion strategies during fight week. About 4–7 g/kg may be suitable for modest carbohydrate restriction. 15. Post weigh-in, rehydration/refueling protocols should aim to regain ≥10% of body mass to mitigate declines in performance and the negative effects of rapid weight loss. 16. The long-term effects of frequent weight cuts on health and performance are unknown, necessitating further research.

## 1. Methods

This position stand focuses on nutritional recommendations that are specific to MMA and combat sports. A comprehensive literature review was conducted using three databases: PubMed, MEDLINE, and Google Scholar. The following combination of search terms were used for each individual database: combat sports, acute weight cut strategies, bioenergetics, fiber depletion, heat acclimation, water loading, glycogen status, hydration, sports nutrition, and nutrient timing. Articles were included if they were peer-reviewed and/or original research. Although few published studies address the complexities and unique training demands of combat sports, relevant articles were selected, regardless of any methodological limitations. The studies were categorized based on the core phases of combat sports: general preparation/off camp, fight camp, fight week, and rehydration/refueling.

## 2. Introduction

Combat sports have a long history, yet organized scientific study is still in its infancy. Several combat-related sports can be traced back to the ancient Olympic games: including armored foot races, boxing, wrestling, and throwing events. These combat sports known as “pankration” entered the 33rd Greek Olympic Games circa 688 BCE [1]. Over the centuries, various disciplines evolved from the ancient art of pankration, such as wrestling, judo, kickboxing, Tae Kwon Do, Muay Thai, and now two of the world’s more popular combat sports: Mixed Martial Arts (MMA) and Brazilian jiu Jitsu. The recent growth and global interest in combat sports and MMA can be largely attributed to the establishment of the Ultimate Fighting Championship (UFC) in 1993 [2]. Currently, in its 31st year, the UFC is estimated to generate over a billion dollars per year [3,4]. Furthermore, the UFC has facilitated the growth of numerous other MMA organizations and has also increased interest in other combat sport disciplines like Brazilian jiu-jitsu [5]. With the increasing popularity of the sport, higher participation rates, and greater earning potential, coaches and fighters are motivated to improve skill acquisition and enhance their physiological capacities. Recognizing the needs of modern combat athletes, the UFC Performance Institute was established specifically to offer evidence-based services in strength and conditioning, testing, nutrition counseling, weight cut supervision, post weigh-in rehydration and refueling strategies, and post-fight recovery.

The intent of this position statement is to review existing literature on combat sport disciplines, with a specific focus on identifying best practices for general preparation, fight camp/longitudinal weight descent, fight week, post weigh-in recovery, and post-fight health and well-being. While there is ample research available on optimal dietary practices for athletes in various sports, there is a noticeable lack of data specifically tailored to combat sports. This is particularly true when it comes to determining the most effective practices for longitudinal weight descent during fight camp, fight week preparation, and post weigh-in recovery. As a result, this position statement places special emphasis on mixed martial arts (MMA), where combat athletes have a pre-day weigh-in. Consequently, the recovery period after a weigh-in can range from 24 to 36 h before the fighter enters the cage, mat, or ring. However, consideration is also given to sports, such as Brazilian jiu-jitsu, and wrestling, where weigh-ins occur less than 24 h before or on the same day as the competition, often with only a 1–2 h window between stepping off the scale and competing on the mat.

## 3. Professional overview

Combat sports and MMA present numerous variables to consider when formulating nutritional strategies, including both foreseeable and unforeseen factors. For example, the bioenergetics and energy requirements across MMA and combat sports disciplines are largely commensurate with the phases of preparation, and the athletes’ total body mass. Factors such as weight class, training styles, volumes, and intensities, may necessitate individualized adjustments with dietary planning. The preparation requirements, duration of competitions, and weigh-in regulations will vary across fight disciplines [6]. In MMA, exceedingly high training volumes are necessary due to the interdisciplinary nature of the sport, where different styles must be trained concomitantly [3]. MMA fighters must possess sound techniques in punching, kicking, wrestling, jiu jitsu, and Muay Thai. This leads to potential increases in training volume and intensities, which in turn influence energy and recovery demands. It is essential to differentiate between the bioenergetics and energy demands of training for competition vs. the competition itself. For example, at the professional level, most MMA fights consist of three rounds lasting 5 min each, with a 1-min rest interval between rounds. In contrast, fight preparation can involve four to 5 h a day of intensive training [3].

It is also critical to consider that nutritional strategies during camp may need to be managed according to the weigh-in regulations of the sport (Table 1). In general, both amateur and professional MMA, boxing, kickboxing, and Olympic judo offer 24-to-36-h windows between the weigh-in and the time the athlete enters the cage, mat, or ring. Most national, international, and Olympic competitions (e.g. wrestling and Brazilian jiu jitsu) require the athlete to weigh-in less than 24 h before a match, or on the same day, often with only 1–2 h to recover before competition. These weigh-in windows will require different methods for each athlete to reach their competitive weight. MMA, boxing, and kickboxing emphasize reducing body fat and relying on rapid weight loss and acute water loss techniques in the final 24–48 h before the weigh-in. This is immediately followed by replenishing fluids, electrolytes, and an emphasis on carbohydrate intake in the subsequent 24–36-h window. In contrast, other combat sports have a smaller recovery window of 1–2 h. This is an extremely important differentiating factor as those combat athletes with a 1–2-h recovery time will not be able to deploy the same type of acute water loss strategies and rehydration practices as those with longer recovery windows. Hence, athletes competing with same-day weigh-ins such as wrestling or jiu jitsu may need to have a greater reliance on the loss of body fat and regulation of lean tissue mass to qualify for their competition as the window for rapid weight loss, acute water loss and rehydration, is limited. Nutritional and weight cut strategies are reviewed throughout this paper with a specific focus on practices for those disciplines with a longer weigh-in and recovery time such as MMA. However, the following considerations are addressed for those with combat disciplines with different post-weigh-in recovery times (Table 1).

**Table 1.** Weigh-in times for Combat Sports.

Sport/Discipline	Competition Rounds	Weigh-In Window and Allowance?
**Weigh-ins < 4 hours before competition**		
Wrestling NCAA	1× 3 minute Periods 2 × 2 minute Periods	Net 0–3% BM loss from sweat losses starting in a euhydrated state.
Olympic Freestyle Wrestling	2 × 3 minute Periods	
IFBJJ	1 × 10 minute Round	
ADCC	1 × 6 minute Round 1 × 8 minute Round in Title Match	
**Weigh-ins within 4–12 h before competition**		
Amateur Boxing	3 × 3 minute rounds	Net 2–4% BM loss from sweat losses starting in a euhydrated state.
Amateur Muay Thai	3 × 2 minute rounds	
**Weigh-ins within 12–24 h from competition**		
IJF Judo	1 × 5 minute Round	Net 3–5% BM loss from sweat losses starting in a euhydrated state.
Olympic Boxing	3 × 3 minute Rounds Men	
Olympic Tae Kwon Do	3 × 2 Minute Rounds	
**Weigh-ins within 24–36 h from competition**		
Professional MMA	3 × 5 minute Rounds or 5 × 5 minute Rounds for Title Fight	Net 4–6% BM loss from sweat losses starting in a euhydrated state.
Professional Boxing	Professional Boxing: 12 × 3 minute Rounds for Title Fights 8–10 Rounds for non- Title Fights	
Professional Kickboxing	3 × 3 minute Rounds- 5 × 3 minute Rounds for Title	
Professional Muay Thai	5 × 3 minute Rounds	

## 4. Bioenergetics

Three energy systems intersect to initiate and regulate the generation of adenosine triphosphate (ATP): the adenosine triphosphate and phosphocreatine (i.e. ATP-PCr or phosphagen) system, the glycolytic (lactic acid or fast glycolysis) system, and the oxidative systems (i.e. aerobic or oxygen) [7]. The ATP-PCr and glycolytic systems are considered anaerobic, while the oxidative system is known to be aerobic. The ATP-PCr system is metabolically powerful due to the rapid transfer of energy in a brief time frame. The glycolytic system provides energy through the body’s digestion of carbohydrates; however, this process is more metabolically costly. Lastly, the oxidative energy system utilizes oxygen to break down carbohydrates, fats, and, in times of high metabolic demands or low energy availability, proteins. Each of these energy systems offers a differing amount of energy release and is intensity dependent [7]. Recognizing the role of each energy system in a specific sport is essential for adequately preparing athletes to optimize their performance during competitions through effective dietary practices, supplementation, training, and recovery protocols see Table 2 [8].

**Table 2.** Energy system production in skeletal muscle for combat and MMA specific skill applications.

Energy System	Energy Contributions	Duration	Skill Applications
ATP/PCR Alactic system Phosphagen (anaerobic)	Immediate energy source skeletal muscle stores as ATP and Phosphocreatine	Initiates explosive activity for 5 seconds to maximum of 10 seconds	Takedown and drive through opponent 4–5 rapid punch combo followed by rear leg round kick
Glycolysis/Lactate Sometimes referred to as Fast Glycolysis	Immediate breakdown of glucose for ATP-production Unsustainable and produces hydrogen ion accumulation	High intensities sustained for 6–30 seconds	Grappling scrambles Chain wrestling moves. Multiple 4–5 strike, intermittent intensity combinations
Oxidative Phosphorylation (aerobic)	Slow rate of ATP production from oxidation of Glucose and Fatty acids- sustained energy production	Aerobic respiration contributes energy for sustained skill application from approximately 2 minutes up to 25 minutes in an MMA title fight or 36 minutes in a boxing title fight	Used in recovery between anaerobic bursts, Recovery between rounds Circling the cage or ring and higher contributions as fight duration increases
It should be noted that energy systems overlap and work in concert, yet a system may dominate in energy contribution dependent on the duration and intensity of the applied skill			

Adapted from: Bounty, P., Campbell, B., Galvan, E., Cooke, M., & Antonio, J. Strength and Conditioning Considerations for Mixed Martial Arts. Strength and Conditioning Journal 33(1): p 56–67, February 2011. | DOI: 10.1519/SSC.0b013e3182044304

Combat athletes not only require elevated levels of cardiorespiratory endurance for sustained performance, but also need explosive knockout power for striking, as well as the necessary strength for effective takedowns and manipulation of their opponents [9]. Determining the exact energy systems and fuel mixtures in real-time is challenging as attaching accurate metabolic measuring devices to the athlete is likely to hinder performance or pose a risk of injury. Simulated fight scenarios in a laboratory setting partially address this issue through the measurement of effort-pause ratios. By using effort-pause ratios, one can infer the physiological and metabolic demands of the fight and differentiate between combat athletes with varying training levels and fighting styles. Research conducted in simulated fight bouts across different fight disciplines has provided valuable insights into the energy systems and substrate utilization.

### 4.1. Mixed Martial Arts (MMA)

MMA requires extremely high energy metabolic demands, and it does so across all three energy systems. The sport may be best categorized as “a high-intensity intermittent sport in which force must be repeatedly exerted against an external resistance in the form of an opponent” [3].

The bioenergetic demands of MMA require managing the mixing of the alactic, fast glycolytic, and aerobic energy systems. Previous work comparing high-level and low-level martial artists showed that successful grappling- and striking-focused athletes have greater anaerobic energy system(s) efforts [10–15]. The variations in anaerobic output (ATP-PCR vs Glycolytic) between strikers and grapplers underscore the differences in work-rest ratios inherent in their respective sports, but other factors such as the pace of a fight/match could exert influence. In striking combat sports, Muay Thai and kickboxing exhibited work-to-rest ratios of 2:3 and 1:2, respectively, which aligns with the characteristics of the ATP-PCR energy system [16]. In grappling sports, such as judo, a greater work-to-rest ratio has been measured at 3:1, which is suggestive of the glycolytic energy system [17]. Similarly, a 3:1 ratio was identified in Greco-Roman wrestling [18]. The work-to-rest ratios of both striking and grappling sports rely heavily on the anaerobic system for high-intensity periods while oxidative pathways contribute to the recovery of anaerobic activity. The larger work-to-rest ratio seen in grappling sports explains the longer-term and longer-bouts of anaerobic efforts seen among high-level grapplers [19,20]. The work-to-rest ratio in MMA has been measured between 1:2 and 1:4 (excluding intervals between rounds), which is midway between striking and grappling sports and displays a combination of both ATP-PCR and glycolytic anaerobic energy system contributions [21,22]. While bouts lasting 15 to 25-min demand a heightened level of cardiorespiratory fitness (i.e. oxidative energy system), MMA athletes attempt to deliver impactful strikes or engage in grappling and wrestling which may necessitate a superior degree of strength, power, and muscular endurance. In other words, the outcome of a fight in MMA can be decided by the ability to optimally engage in intermittent bursts of maximal exertion within brief time frames.

In title fight bouts, MMA athletes compete for a maximum of 25 min (5 × 5-min rounds). Each round may consist of high-intensity periods of action lasting from 6 to 36 s [23]. This is combined with episodes of lower-intensity action that may last two to three times as long. Notably, however, knockouts and TKOs occur in these high-intensity areas of activity, making these explosive efforts critical to success [3]. Moreover, the MMA athlete’s metabolic capacity may be influenced by factors such as the demands of the weight cut, post weigh-in recovery, the pace of the fight, the fighting styles, and which opponent is dictating the style and pace of the fight. Hence, numerous factors will influence the energy requirements of MMA training or competition. No studies to date have accurately measured the caloric expenditure of full-training sessions or a mock three-round fight. Research across various combat sport disciplines has mostly focused on Olympic sports. Although the translation of findings from other combat sport disciplines may not offer exact parallels to the bioenergetic demands of an MMA match, it does offer valuable insights into the caloric expenditure and distribution of energy system utilization.

### 4.2. Karate

During the 2020 Olympic Games, karate was one of the striking sports that was represented [24]. Beneke et al. [25] used excess post-exercise oxygen consumption (EPOC) and blood lactate measures during a simulated fight to analyze karate athletes. After 36 matches, consisting of 2–4 fights per athlete with a mean duration of 275 ± 61 s and 9-,15-, and 17-min intervals between matches, it was demonstrated that these fights had 77.8 ± 5.8% contribution from the oxidative system, 16.0 ± 4.6% from the ATP-PCr system, and 6.2 ± 2.4% from the glycolytic system. The total observed energy expenditure was 81.4 ± 19.5 kcals. These findings suggest that during a karate fight, athletes rely predominantly on oxidative metabolism while still receiving support from anaerobic systems. Beneke et al. [25] concluded that athletes who were most efficient at utilizing the ATP-PCr energy system were the most effective at executing actions during the fight and those who maintained glycolytic power throughout the fight made the most offensive actions. In agreement, Doria et al. [26] also showed that the oxidative system (74 ± 1%) was the predominant energy system in karate. The researchers observed that the ATP-PCr system contributed 14 ± 3% and the glycolytic system contributed 12 ± 2% during a karate match, with a total expenditure of 72.8 kcals ±6 kcals. During this study, they also compared sex differences and found that males relied more on oxidative contributions when compared to females.

Loturco et al. [27] completed a case study on a male karate athlete, who is a double world champion, the athlete performed a simulated karate bout. Their findings again aligned with the previous work of Beneke and Doria in that they reported an energy expenditure of 88.5 kcals, 31% of energy production was derived from the glycolytic system while the oxidative system contributed 61%.

### 4.3. Taekwondo

Campos et al. [28] reported a 66 ± 6% contribution from the oxidative system, 30 ± 6% contribution from ATP-PCr, and 4 ± 2% from the glycolytic system in taekwondo athletes during a simulated match. In each round, there was a mean expenditure of 43 ± 7 kcal. In addition, the contribution of energy systems varied in each round. In round one, the oxidative system (23 ± 3 kcal) compared to round 2 (30 ± 3 kcal) and round 3 (32 ± 4 kcal). The contribution from the glycolytic system also decreased from the first round (2.6 ± 0.9 kcal) to the third round (1.5 ± 1.1 kcal). The first round required less energy expenditure (38 ± 4 kcal) compared to the second (43 ± 4 kcal) and third (49 ± 7 kcal) rounds. To maintain the same level of technical action, the athletes expend more energy in the later rounds than in the first, which indicates a decrease in efficiency. The only overall change in energy systems was a lower contribution from the glycolytic system in the first round (7 ± 2%) versus the last round (3 ± 3%). The ATP-PCr system had an equal contribution across all three rounds, indicating that the 1-min interval of rest was sufficient for replenishing phosphocreatine stores. The high energy demand in taekwondo is mainly met by the ATP-PCr system, which provides energy to sustain repeated striking attacks involving both the upper and lower body [28].

### 4.4. Judo

Julio et. al. [29] examined the energy system contribution in grappling sports. During this study, the authors reported that the oxidative system contributed 70%, while the ATP-PCr system contributed 21% and the glycolytic system provided 8% of the energy needed during the match duration. Their total energy expenditure was 84 ± 22 kcal. By assessing the onset of blood lactate accumulation (OBLA) and VO_2_Peak for upper and lower body cycle ergometry, they observed some shifts in metabolic systems. During a match, the energy consumption of a judo athlete appears to be between their VO_2_Peak and their OBLA. The researchers found a lower oxidative system contribution in 1-, 2-, and 3-min matches compared to those of longer duration. The shorter matches showed a greater reliance on the ATP-PCr system, and longer matches used greater oxidative contributions. The glycolytic system remained constant throughout the length of the matches. Power was seen to be notably higher while rest time was lower in the 1-min matches compared to the longer matches. This indicates that nutritional and training interventions should be aimed at maintaining metabolic power over time to improve performance.

### 4.5. Boxing

Davis et al. [30] simulated an Olympic boxing match consisting of three rounds, each lasting 3 min. They showed that the oxidative system contributed 86% to energy production while ATP-PCr contributed 10%, and the glycolytic system contributed 4%. The boxers were reported to be at 97–100% of their VO2Peak during the last 20 s of each round. The contribution of the oxidative energy system was lowest during the 1st round (30 ± 4.8 kcal) compared to the 2nd and 3rd rounds (33.8 ± 5.7 kcal and 33.6 ± 6.8 kcal, respectively), while the glycolytic system was highest during the 1st round (3.2 ± 0.9 kcal) with concomitant decreases for the 2nd and 3rd round (1.9 ± 0.6 kcal and 1 ± 0.8 kcal, respectively). Interestingly, the boxers were able to maintain the same total number of offensive attacks per round. The authors suggest the energy demands are slightly lower compared to a competitive bout which would shift metabolic demands to slightly more anerobic but still predominantly aerobic. However, Davis et al. [30] concluded that these physiological and energy system responses to the boxing match indicate that the athletes were metabolically efficient, and they exhibited a high level of aerobic fitness, as seen by their ability to supply ATP through oxidative pathways rather than glycolytic flux during the rounds.

### 4.6. Muay Thai

Crisafulli et al. [31] examined ten experienced competitive Muay Thai athletes during a simulated match consisting of three rounds (3 min per round) with a 1-min rest period between rounds. This is consistent with many amateur Muay Thai bouts. The investigation showed that the average heart rate and oxygen uptake for all athletes were above those respective values when evaluated at lactate threshold for all three rounds. However, even though the heart rate and oxygen consumption slightly decreased during the 1-min period between rounds, they remained above the lactate threshold and thus did not allow for full recovery. The athletes’ efforts beyond levels of aerobic metabolism produced increases in CO_2_ production because of the body’s shift toward anaerobic processes, which is intricately linked to the observed rise in blood lactate. This indicates a meaningful relationship between the two. This also served as an indirect index of glycolysis. The researchers showed that after an initial increase, the excess CO_2_ peaked in the first round and subsequent rest period, and there was a continual increase on the reliance of the aerobic system. Therefore, glycolysis predominated early in the match and then progressively waned as the contribution of the aerobic system increased in the following rounds [20].

### 4.7. Jiu jitsu/wrestling

Wrestling, submission grappling, and Brazilian Jiu-Jitsu are commonly integral parts of an MMA match, yet this can be contingent on the disciplines and styles of the fighters competing. The sport of wrestling is likely to place a significant demand on all energy systems. Ohya et al. [32], purported that the technical and tactical requirements of wrestling, coupled with the muscle-strength andpower demands on both the upper and lower body, foster the use of both the anaerobic and aerobic energy systems. The anaerobic system provides the quick bursts of maximal power during the match, whereas the aerobic system contributes to the wrestler’s ability to maintain effort for the duration of the match and to recover between periods [32].

A study by Mirzaei et al. [33] investigated the energy systems in Greco-Roman and Freestyle wrestlers participating in the 2015 and 2016 World Championships. Energy contributions remain comparable across weight classes in both the Freestyle and Greco-Roman wrestlers [33]. The study found that glycolytic/lactic acid system contributed the most in energy release during a single wrestling match, which represents the anaerobic nature of wrestling. Additionally, the results showed that the nature of Freestyle is more dynamic than Greco Roman, because Freestyle wrestlers can use all the Greco-Roman techniques as well as leg attacks by using arms and legs actively. Indeed, Kraemer et al. [34] reported that large increases in lactate (up to 20 mmol/L) occurred during a five-minute college freestyle match, which is associated with reductions in power production and fatigue. In contrast, the authors of this review cited that maximal treadmill tests only raise lactate to approximately 10 mmol/L.

However, factors such as the duration and intensity of the match, skill level, level of competition, and movement efficiency may significantly influence the relative contribution of each system. Accordingly, determining the precise contribution of each energy system in wrestling matches remains challenging.

A recent study by Pessoa Filho et al. [35] investigated the metabolic demands of a single six-minute no-gi sparring match with 10 advanced no-gi grapplers. Their investigation reported approximately 28% of energy contribution was derived from the ATP-PCr, and approximately 72% was derived from anaerobic energy contributions. While the investigators did not measure aerobic contributions, they asserted that matches of sustained lengths would have a greater reliance on aerobic metabolism. The authors also noted that differences in the style of an individual jiu jitsu competitor, the level of competition, perceived stress, and opponent effort, potentially influenced energetic pathway utilization.

#### 4.7.1. Bioenergetics summary

In summary, combat sports involve high-intensity actions that are metabolically demanding, so athletes must have good metabolic flexibility and efficiency. Research on martial arts has generally found that the energy demands of an average karate fight lasting 4 min and 27 s come from aerobic (77.8%), anaerobic alactic (16.0%), and lactic (6.2%) energy pathways [25]. Moreover, the existing research is consistent; independent of the combat sport discipline, as the duration of the match increases, the contribution of the aerobic system increases, yet anaerobic alactic pathways remain vital for executing high output bursts of skill applications. It is important to note that sports nutrition professionals should be aware of the numerous factors that can influence energetic demands, energy expenditure, and ultimately metabolic cost. With an understanding of the complex interaction of energy systems, sports nutrition professionals can effectively manage the total daily energy expenditure requirements based on the combat athletes training volumes, intensities, and body composition, and adjust the calories and macronutrients, as necessary.

## 5. Combat sports – preparation phases

As noted, combat sports are a group of contact activities in which fighters engage in one-on-one physical contests depending on a specific set of rules for competition. Combat athletes can strike, kick, hit, throw, punch, or grapple with their opponent, depending on the ruleset, with the aim of trying to score more points by disabling their rivals and stopping them from continuing the match [36]. The physical stresses of combat athletes are highly individualized and vary based on the demands of their training specialty (jujitsu/boxing/grappling), weight class, and the timing of the next match. Phases of combat sports are generally separated into three distinct phases: “Off camp” or general preparation, “fight camp” and “fight week”/post weigh-in. General preparation is commonly referred to as “off camp.” As the name implies, this phase occurs between fights and outside of a formalized fight camp. This phase should focus on improving aerobic capacity, advancing skills, developing strength and power, and maintaining an appropriate body mass and body composition. Fight camps, on the other hand, are eight-to-ten-week periods of time where the fighter works extensively on advancing multidisciplinary skills that target tactical strategies, and specifically advancing the requisite bio-motor qualities needed to be successful in competition. This often involves two to three daily training sessions, which are done commensurate with the longitudinal weight descent. Finally, fight week requires the fighters, coaches, and sports nutrition professionals to strategically manage skill sessions and manipulate nutritional and weight cut strategies, which include adjusting caloric intake, carbohydrate reductions, and water loading. Additionally, fighters utilize acute water loss strategies such as dry sauna, hot water immersion, and mummy wraps. At the professional level, many of these practices during fight week must be managed with numerous press and media obligations.

### 5.1 Off camp/general – preparation phase

The “off camp” phase occurs between fights and precedes the formalized fight camp. This phase, which can vary in length, should focus on advancing aerobic capacity, technical skills, developing strength and power, and maintaining an appropriate body mass and body composition.

#### 5.1.1. Body mass and weight

Combat athletes may benefit from maintaining adequate levels of aerobic fitness along with managing their body mass and body composition during the general preparation phase; especially as it pertains to health and performance. In this respect, an athlete’s “walk-around weight” is an important metric outside of fight camp. Walk-around weight is generally defined as the athlete’s weight outside of the fight camp measured against their weight class. It is estimated that combat athletes lose an average of 5% body mass during rapid weight loss [37]. Garthe et al. [38] reported minimal losses in performance measures (1-repetition-maximum (1RM) tests, 40-m sprint, and countermovement jump) in individuals engaged in weight loss at rates of 0.7% per week. Based on typical loss during RWL and weekly body mass loss of 0.5–1% over an 8-week camp, the authors suggest that the ideal maximum walk-around weight should range from 12% to 15% above the athlete’s desired weight class. The authors speculate this will minimize decreases in performance while allowing the athlete to make the weight of their desired class. In context, this would have a UFC male middleweight, required to make 185Ibs for their weight division, sustaining a weight of 207Ibs–212Ibs in the off camp phase. However, these numbers have not yet been fully substantiated through research and while there is scant research on this notion, practical application suggests this range should be effective for a longitudinal weight descent that simultaneously supports conditioning, skill training, and recovery. During fight camp, the athlete will utilize dietary and exercise strategies that create a caloric deficit to decrease body mass and must ensure this deficit does not negatively impact training performance, overall health, and any potentially harmful long-term metabolic adaptations. Although 12–15% may be potentially optimal, larger reductions in body mass are common practice in combat sports [39].

Moreover, research has shown large decreases in testosterone below clinical reference ranges (<5 mmol·L^−1^), as well as a decrease in resting metabolic rate, when losing 13–15% body mass over an 8-week camp [40]. Accordingly, the ranges of 12–15% body mass above the weight class may be the upper limits of a suitable longitudinal weight descent.

Combat athletes exceeding 12–15% body mass will require more significant energy restriction, and if completed over a shorter duration than 8 weeks, the caloric deficit needed to achieve the desired changes in body mass may put athletes at a greater risk for low energy availability. While much of the available literature on low energy availability has indicated systemic reductions in various hormones, the impact of these energy deficits may also result in negative changes related to skill acquisition, concentration, motivation, and recovery [41]. This is a critical area for future research, which aims to identify suitable losses in body mass that will allow for sustained longitudinal health and performance for combat athletes.

#### 5.1.2 Energy requirements and macronutrients

Like many other types of athletes, the success of combat athletes is highly dependent on their ability to consume adequate energy to meet the demands of multiple daily training sessions. Daily energy needs are based on the athlete’s body mass, body composition, training volumes, goal weight, and total daily energy expenditure (TDEE). The caloric needs of elite combat athletes engaged in moderate to high-intensity training differ significantly from those of the general population. To maintain weight, athletes must balance caloric intake with total energy expenditure, so determining TDEE with reasonable accuracy is necessary. Metabolic carts and indirect calorimetry are the most accurate methods to determine a combat athlete’s resting metabolic rate (RMR). In the absence of these modalities, total energy expenditure can be calculated using the Mifflin – St Jeor equation, which considers age (years), sex, height (cm), weight (kg), and an estimate of the athlete’s physical activity level (PAL) [42,43]. The Cunningham equation can also be used which determines BMR via, BMR (kcal/day) = 500 + 22 × (lean body mass). The Cunningham equation determines the energy required at rest by considering lean body mass instead of overall body weight and then factoring in the combat athlete’s physical activity level. Another simple estimate of total energy expenditure is to multiply the athlete’s weight (lbs.) by 14 and add the estimated calories burned by training and the thermic effect of food [43], which is typically 3% to 10% of calorie expenditure [44]. These methods provide a reasonable estimate for caloric expenditure which should then be adjusted to best match the combat athletes’ current body mass, training demands, and for the maintenance of a desired weight and body composition.

Combat athletes meet their caloric requirements by consuming adequate amounts of carbohydrates, protein, and fat. Carbohydrate requirements vary significantly based on body mass, the type of workout, training intensity, and the training phase in which they are engaged [43]. Generally, combat athletes should anticipate consuming between 3 to 5 g of carbohydrates per kg of body mass daily for light activity and between 8 to 12 g of carbohydrates per kg of body mass daily for intense training [43]. Although research suggests 8–12 g of carbohydrate per kg of body mass may be required for endurance athletes, this amount is likely to be excessive for combat athletes competing in a weight-specific sport. However, one should not overestimate the merit of consuming these amounts for post weigh-in refueling. Fruits, vegetables, whole grains, sweet potatoes, rice, pasta, and legumes are recommended sources of carbohydrates during the “off camp” phase, instead of processed sugars [43].

Fat requirements for the combat athlete are the same as nonathletes and should be 20–35% of total daily calories or approximately 1.0 g of fat for every kilogram of body mass per day. While being the most energy-dense nutrient, dietary fat is a common target for people who need to restrict energy intake; however, consuming daily fat intake below 15–20% of total calorie intake is not advised [45]. However, fats are often restricted during longitudinal weight descents to meet the restriction in calories while preserving carbohydrate and protein intakes. Optimal fat sources include salmon, lean meats, eggs, nuts and nut butters, avocado, coconut, and olive oil [43], and not saturated or trans fats from fried foods, processed cheeses, or hydrogenated oil.

Protein requirements range from a minimum of 1.2 g per kg of body mass per day and up to 2.4 g of protein per kg of body mass or 15% to 30% of total calories [43] which is a higher intake relative to the average sedentary individual [46,47]. In addition to promoting muscle recovery and enhancing muscle protein synthesis, increased protein intake results in favorable changes in body composition, especially when in an energy deficit. It is imperative to meet protein requirements since, unlike fats and carbohydrates, there is a limited storage pool of proteins and their constituent amino acids. If combat athletes are not meeting protein requirements via sufficient food intake, protein supplementation in the form of protein powders or ready to drink beverages might be needed to achieve adequate energy and protein needs. The sports nutrition professional can assist combat athletes in building proper meals that meet energy and macronutrient demands, with strategically timed windows around their training sessions.

Although micronutrients are considered essential for optimum health, their ability to function as ergogenic aids is unlikely [43], mainly when overt deficiency was present before vitamin and mineral supplementation. Nonetheless, studies have highlighted that consuming a multivitamin can help athletes meet their daily micronutrient needs, which are higher compared to the general population [48].

#### 5.1.3. Periodized nutrition

Another emerging area of research is “periodized nutrition,” which refers to the strategic use of specific nutritional interventions to support exercise performance [49]. This is not a new concept. It is well established that protein should be consumed post-exercise, as this can optimize net protein synthesis. However, most nutritional recommendations for athletes aim to promote acute recovery after exercise and are not specific regarding the type of training or intensity [49]. Pairing specific nutritional goals commensurate with the phases of training may be beneficial for the combat athlete whose nutritional needs extend beyond recovery [49]. For example, protein needs should be based on increasing or maintaining a suitable body composition in the general preparation phase. Moreover, protein needs should be tailored to recovery and minimizing loss of lean body mass during fight camp. The authors recommend creating individualized nutrition and training programs, ideally before the start of fight camp, and adjusting as needed.

#### 5.1.4. Assessment

Prior to entering fight camp, it is important to conduct a thorough assessment of the specific demands of the combat sport discipline and the individual athlete’s needs. An assessment enables the training team to determine which nutritional strategies should be implemented for the combat athlete and what needs to be achieved before fight camp. Information such as the athlete’s anthropometrics, previous weight cut practices, the number and duration of weight cuts, and current dietary intake (including supplementation) should be assessed. Daily food logs or dietary tracking apps are accessible to most individuals and are ideal for assessing and monitoring dietary intake. The combat athletes’ previous practices can be measured against an evidence-based approach and revised when warranted.

While there are numerous measures that may have utility in optimizing performance through nutritional interventions. The sports nutrition professional may be most concerned with data including resting metabolic rate (RMR), body composition including fat-free mass (FFM), body fat percentage, and total body water (TBW). First, this data may assist in developing nutrition strategies that will meet the energy demands for combat athletes through all phases of training. Additionally, via determining total body mass and fat-free mass, predictive equations can be used to establish the feasibility of the combat athlete competing in their weight division. While there are no specific validated equations for predicting the efficiency and safety of longitudinal weight descents, the UFC Performance Institute has developed the Weight Making Preparedness Score (WMPS). The equation requires data on Fat-Free Mass, the athlete’s Total Mass over the target weight, Fat Mass, and Resting Metabolic Rate. The equation can be found on pp. 242 of the Cross-Sectional Performance Analysis of the UFC Athlete, vol. 2.

This data may also have utility for determining the required rate of a longitudinal weight descent in fight camp; and for monitoring any potential deviances in resting metabolic rate. Accordingly, the sports nutrition professional may track these measures (body composition and resting metabolic rate) and adjust nutritional strategies as needed to ensure adequate energy intake and an efficient longitudinal weight descent.

There are various methods available for testing body composition, which can be used by researchers and trainers. Common methodologies for measuring body composition include hydro-densitometry, air displacement plethysmography, multifrequency bioelectrical impedance analysis (MF-BIA), ultrasound, 3D scanning, skinfold thickness, and dual-energy X-ray absorptiometry (DXA) [50] Each method has its own strengths and weaknesses, as assessed by researchers. Dual-Energy X-ray Absorptiometry (DXA) is considered one of the most accurate methods for assessing body composition. DXA provides information on total and regional fat mass, fat-free mass, percent fat, and bone mineral density [51]. Additionally, DXA can provide limb-specific estimations of fat mass and fat-free mass, which is useful for measuring and tracking injury/recovery and fat loss for weight cutting [52,53]. Essential DXA measurements for combat athletes include body composition analysis, muscle-fat analysis, and obesity analysis. It should be noted that DXA, as with all modalities, is subject to confounding results from inter-assessment differences in hydration, glycogen and muscle creatine levels, all which can vary significantly in combat athletes [54].

Bioelectrical impedance analysis (BIA) is an inexpensive and reliable assessment tool used for body composition. Unlike DXA, BIA estimates total body water, which can be useful for estimating the impact of fluid loss on pre- and post-training and pre- and post-weight-in. Important BIA measurements to consider in combat athletes are body composition analysis, muscle-fat analysis, and obesity analysis. Compared to DXA, BIA has been shown to underestimate fat mass and overestimate fat-free mass [51,55–60]. Despite this variability in findings with BIA, it can still be considered a useful tool for measuring body composition change, which is especially important for sports nutrition professionals assessing directional change [51,55,61].

As part of a comprehensive assessment, when possible, blood analysis screenings may provide insight into a combat athlete’s health and performance. Blood work is often used as a screening tool to identify potential deficiencies in vitamins, minerals and microminerals such as iron, as well as evaluating oxidative stress and inflammation. Additionally, blood work can provide the status of red blood cell populations as well as markers such as cortisol and creatine kinase, which can provide insights into overtraining, readiness to train, and other measures such as protein turnover [62]. Biomarkers such as vitamin D and iron are well established and commonly assessed, whereas others need further research (e.g. fatty acids) to demonstrate their utility in sport [62]. Newer methods that estimate plasma volume using groups of biochemical markers are also showing promise. Such markers may provide additional support for monitoring a combat athlete’s adaptation to training.

#### 5.1.5. Performance supplementation

Supplementation with various dietary ingredients is a widespread practice for many athletes to heighten performance and augment training adaptations. While research on combat sport athletes is limited, some evidence is available which suggests certain ingredients may enhance their performance. The phase of training or preparation whereby an athlete may choose to utilize a certain dietary supplement is an important consideration. For many supplements, utilizing them during a general preparation phase is a prudent consideration. This allows for experimenting with a given supplement and the dosing to optimize the effects on the combat athlete. Some supplements, however, rely on their accumulation in skeletal muscle (e.g. creatine, beta-alanine, etc.) to exert their ergogenic effects. In these situations, the off-camp/general preparation phase allows for a sufficient window of time for these supplements to reach ergogenic levels. Notwithstanding these considerations, the following supplements may be considered for their ability to augment performance and recovery potential of combat athletes: Caffeine [63], beta-alanine [64], sodium bicarbonate [65], creatine [66], and nitrates [67,68]. In addition, supplements such as essential amino acids [69], leucine [70], [71], branched-chain amino acids [72–74], and β-Hydroxy β-methylbutyrate [75], may promote increases in protein synthesis or decrease delayed onset muscle soreness.

Caffeine is a long-standing supplement in the realm of athletic performance. Caffeine use has been shown to improve physical and cognitive performance in several sports due in large part to its ability to modulate central and peripheral nervous system activity [63]. Several studies support the use of caffeine among combat athletes [76–78]. A systematic review conducted by Lopez-Gonzalez et al. [79] found that supplementation with 3–6 mg/kg of caffeine increased the glycolytic contribution (as evidenced by increased blood lactate concentrations) to energy metabolism during the execution of real or simulated combat bouts. Caffeine supplementation also improved levels of strength, power, and upper arm muscular endurance, and these effects were not associated with an increase in the perceived exertion by the athlete [80].

A systematic review by Lopes-Silva & Franchini in 2021 [81] examined the isolated and combined effects of sodium bicarbonate (NaHCO_3_) and β-alanine (BA) supplementation on the performance of combat sports athletes. The results showed that acute and chronic isolated ingestion of NaHCO_3_ and chronic BA are effective in improving performance in the Wingate test, Upper Wingate test, Special Judo fitness test and Karate-specific aerobic test, which translates to advancing combat sports athlete’s performance via muscle functionality and localized muscle buffering capacity. Additionally, the co-ingestion of BA and NaHCO_3_ resulted in additional improvements across testing. However, the authors found no benefit to taking these supplements individually.

Halz et al. [82] conducted a study with 16 elite judo athletes (21.8 ± 2.5 years old) where athletes were randomly assigned to receive either BA (4 g/d over the first 2 weeks and 6 g/d in the last 2 weeks) or placebo for 4 weeks. Before and after BA supplementation, the athletes completed two double 30-s upper and lower limb Wingate tests, separated by 3 min. The results showed that BA supplementation significantly improved lower and upper limb total work and upper limb mean power during the Wingate test. However, there were no significant differences in lower limb mean power in the BA group when compared to the placebo group. These authors concluded that chronic BA supplementation effectively enhances high-intensity intermittent upper- and lower-body performance in highly trained judo athletes.

Creatine monohydrate is one of the most studied supplements in human performance, and its safety and efficacy are indisputable [83,84]. Studies consistently demonstrate positive ergogenic effects on single and multiple bouts of short-duration, high-intensity exercise activities, in addition to enhancing exercise training adaptations after creatine supplementation. These qualities are crucial to success in MMA [85–87]. Another proposed benefit of creatine, which is vital for sustaining the training volumes and intensities associated with combat sports, is the facilitation of glycogen replenishment. Roberts et al. [87] confirmed the ability of creatine supplementation to enhance post-exercise muscle glycogen storage during a conventional and rigorously controlled carbohydrate-loading regimen in humans. Additionally, creatine may also provide benefits for MMA athletes by mitigating the effects of traumatic brain injuries (TBI), and ongoing therapeutic applications are now being examined [88]. Doses ranging from 5 to 10 g/daily are generally recommended for enhancing performance, however research is indicating that higher doses may be warranted for the potential neuroprotective effects.

Delleli et al. [89] conducted a systematic review on the effects of beetroot juice (nitrates) on performance in combat athletes. While this review did not assess the effects of combat sports skills, it showed that nitrates may improve oxidative metabolism and force production for combat sports athletes. Effects may vary based on the population, intake duration, muscle group activated, and exercise type.

β-hydroxy-β-methyl-butyrate (HMB) is a metabolite of leucine that is formed through α-ketoisocaproate. Recently, Tartibian and Rezaei [68] demonstrated that HMB supplementation significantly improves physiological recovery markers, attenuates muscle damage, and enhances recovery by modulating cortisol, IGF-1 levels, and perceived recovery status in elite wrestlers following a simulated wrestling protocol. Durkalec-Michalski et al. [90] demonstrated that HMB supplementation significantly improves body composition and enhances both aerobic and anaerobic capacities in highly trained combat sports athletes over 12 weeks without affecting blood markers [91]. In a study by Hung et al. [92], it was found that short-term oral supplementation of three grams per day of HMB for 3 days can reduce body fat in well-trained female judo athletes undergoing energy restriction, without affecting lean body mass or exercise performance. In support, Chang et al. [93] found that HMB supplementation during acute weight loss in well-trained boxers can preserve fat-free mass and maintain heart rate response during simulated matches, without significantly affecting glucose, fat, and protein metabolism under energy restriction. The exact mechanism of action is not clear, but it is believed that HMB has an anti-catabolic effect (possibly by sparing leucine) and promotes anabolic action [94]. These anti-catabolic effects can be particularly beneficial during fight camp when training volumes and intensities are increased, and caloric intake is decreased for weight-loss purposes. The literature suggests that a dosage of 1–3 g (38–40 mg/kg) of HMB can be beneficial [94,95].

Essential amino acids (EAAs) can play a vital role in fostering muscle protein synthesis in combat athletes [96]. Essential Amino Acids cannot be synthesized de novo in humans and must be supplied by diet. Essential Amino Acids ingestion in free form in approximately ten-gram doses or as part of a protein bolus of 20–40 g after exercise are known to stimulate muscle protein synthesis [97]. From these results, consuming EEAs could increase strength and improve body composition by increasing lean body mass, and preserving lean mass during the longitudinal weight descent of the combat athlete [98].

A well-balanced diet may provide sufficient micronutrients, but combat athletes who practice rapid weight-loss or eliminate whole food groups may be at risk of nutrient deficiencies [43]. Additionally, the high training loads of fight camps and elevated energetic demands are believed to lead to an increase in micronutrient needs [48]. Supplementation with basic micronutrients may be warranted.

The sports nutrition professional may consider these supplements as part of the training practices of combat athletes. Therefore, remaining abreast of the current research will aid the sports nutrition professional in deciding which supplements have the highest efficacy and utility for the combat sport athlete. It is vital that the sports nutrition professional recommend supplements that have been third-party tested to meet label claims and be free of banned substances.

#### 5.1.6. Off camp – general preparation phase – practical applications

- The general preparation phase is a time to improve aerobic fitness, improve skills, and optimize physical qualities such as strength, power, speed, and mobility.
- Anecdotally, combat athletes’ maximum walk-around weight should range from 12–15% above their desired weight class.
- Determining the combat athlete’s resting metabolic rate and TDEE is imperative for maintaining an optimal weight and pairing energy and nutrient demands to training volumes and intensities.
- Combat athlete’s protein requirements range from 1.2–2.4 g/kg, with amounts closer to 2 g/kg being considered a target intake.
- While 8–12 g Carbohydrates/kg body weight has been recommended for athletes completing high volumes of endurance exercise, a range of 4-5 g Carbohydrates/kg body weight may be an appropriate starting intake for combat sports athletes once matched with TDEE in the general preparation phase.
- The general preparation phase is an ideal time to determine the potential supplements needed to support the rigors of training and recovery.
- Supplements supported via evidence, such as caffeine, creatine, beta-alanine, nitrates, and essential amino acids, may be used to support the training demands and recovery for combat sport athletes. HMB may preserve lean body mass during the longitudinal weight descent phase.
- Obtaining a detailed history/assessment of the combat athletes’ previous dietary practices, weight cut strategies and frequency of previous weight cuts will help the sports nutrition professional develop the most suitable nutritional practices for all phases of the combat athletes’ training.
- Determining and assessing body composition, fat-free mass, and resting metabolic rate will enable the sports nutrition professional to develop appropriate nutrition strategies.
- Blood work screening, when accessible, may hold value as part of the assessment process by identifying individual nutritional needs and/or deficiencies.

### 5.2. Fight camp phase

Fight camp refers to the formalized training period leading up to a pre-determined fight date. The average fight camp phase ranges from 8 to 10 weeks, depending on the competition schedule. During this time, combat athletes are preparing for the demands of an upcoming match. Training regimens are characterized by moderate to intense exercise which usually includes strength training, technical sparring, multi-disciplinary skill acquisition, and recovery modalities.

#### 5.2.1. Longitudinal weight descent

The types of weight-loss methods that are utilized prior to competition across all combat sports include: fasting, low energy intake, gradual dieting, restricting fluid intake, increasing sweat response such as heated wrestling, plastic suits, saunas, and heat exposure. Other practices commonly include taking diet pills, enemas, laxatives, and engaging in bulimia [99]. While these tactics are adopted at times, they are not endorsed by the ISSN, and the authors of this position stand. Furthermore, self-reported data show that gradual dieting and increased energy expenditure due to greater training frequencies are common in all combat sports. However, the weight-loss practices are not identical between combat sports, with the most common practices shared between boxing and MMA [99].

**Figure 1.**
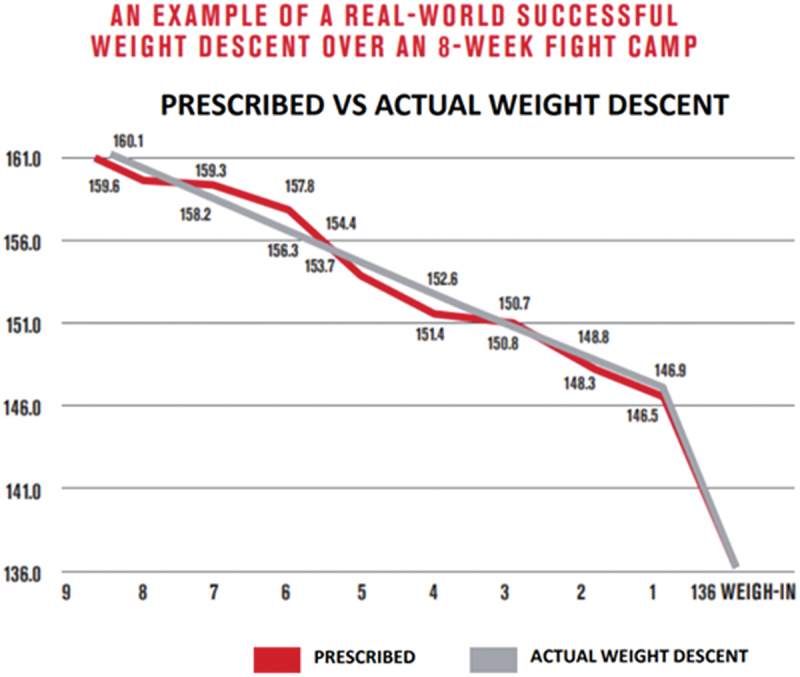
Example of a real-world successful weight descent over an 8-week camp [3].

A primary physiological objective of fight camp is to decrease body fat. Such decreases can be advantageous for combat athletes as it may foster a greater strength-to-mass ratio, improve the endurance needed for mastering skill applications, and lead to a more effective longitudinal weight descent. Moreover, lean body mass holds more water compared to adipose tissue [100]. Therefore, maintaining or increasing lean body mass may aid in successful water manipulation and effective acute water loss strategies 24–48 h prior to weigh-in. Some combat athletes gradually reduce weight during the general preparation phase, which may be warranted if they are above the targeted 12–15% walk-around weight threshold. In this regard, athletes have started their weight descent as high as 18% above their required scale weight and successfully arrived at the weight for their division in 8 weeks as shown in Figure 1. However, this magnitude of weight descent was closely supervised by the nutrition professionals of the UFC Performance Institute and may not be advised for most athletes.

Data provided by: Institute UFC Performance Institute: A Cross-sectional Performance Analysis and Projection of the UFC Athlete. (PI U ed., vol. 2. pp. 485: Zuffa LLC; 2021:485.

An area of emerging research involves investigating the effects of specific diets on athlete performance. The high fat diet in endurance athletes may induce metabolic adaptations [101,102], but it has been debated whether these changes improve performance [103]. In contrast, high carbohydrate diets are shown to increase short-term performance and recovery when performing repeated high-intensity activities and recovery, which is the underlying nature of combat sports [49,104,105]. Higher protein intake during the longitudinal weight descent promotes the preservation of lean body mass during caloric deficits [106]. Although protein is a vital macronutrient, higher protein intake should not compromise appropriate intake of carbohydrates and/or fat intake. Good sources of protein are lean meats such as skinless chicken, steak, eggs, fish, lentils, beans, and isolated or concentrated protein powders [43]. Ready-to-drink protein shakes and powders may have utility during fight camp for use between training sessions, since combat athletes often have several sessions a day.

Combat athletes are commonly recommended to aim for weight loss of 0.5–1 kg of body mass each week with the resulting energy deficit being based largely on total daily energy needs [38,107]. This rate of decline and the concomitant magnitude of energy deficit is contingent upon the length of the camp and how much body mass must be lost. Accordingly, the sports nutrition professional can calculate a rate of weekly weight loss based on the duration of the fight camp and the total loss in body mass that is required; then plan a systematic longitudinal weight descent.

It is advisable that the sports nutrition professional carefully monitor changes in body mass and body composition as it is common for combat athletes to lose weight too swiftly if they are not meeting their energy requirements during fight camp. This may lead to decrements in skill training, endurance, recovery, motivation, and losses in lean tissue [38].

As discussed, the Mifflin St. Jeor or Cunningham equation coupled with the levels of physical activity can be used to calculate energy needs during the longitudinal weight descent phase of fight camp. Although undesirable, if the athlete starts fight camp >15% above their desired weight class or if training camp is of shorter duration, such as <8 weeks, a greater speed and magnitude of weight loss will be required. However, due to the intense training demands during fight camp, carbohydrate intake should not drop below 3–4 g/kg/d [86,98,108]. A protein intake of 1.2–2.2 g/kg/d is advised to preserve lean tissue and aid in recovery during the energy deficit [54,106]. To facilitate an energy deficit needed for a 0.5–1 kg loss of body mass per week, fat intake is often manipulated to elicit a greater energy deficit, resulting in fat intake levels that can range from 0.7 to 1.3 g/kg/d. However, this range may need to be further adjusted to couple with TDEE. As the combat athlete’s body mass descends, subtle adjustments in energy intake may be warranted and most often a decline in energy is commensurate with the loss in body mass and/or changes in RMR that occur. Moreover, this strategy must still support the total training volume, which may include skills training and conditioning.

#### 5.2.2. Tapering

Tapering for MMA competition often involves a strategic reduction in training load while integrating heat acclimation, sweat sessions, and nutritional manipulation to prepare for fight week and competition. The taper period typically begins 1–2 weeks before fight week and may involve a reduction in training volume by approximately 40%. Incorporating high-intensity, fight-specific drills, helps maintain neuromuscular adaptations at an intensity sufficient to prevent detraining effects and maintain technical acuity [109,110]. Additionally, increasing heat acclimation sessions, such as using a sauna or hot bath, can enhance sweat rates, improve heat tolerance, and aid in weight management during tapering period [111]. These sessions, combined with additional sweat suit or plastic-wrapped cardio sessions, may be employed to compensate for the decreased training volume [37].

Nutritional strategies during the taper should involve a methodical decrease in calories and hydration to align with the reduced training load and manipulating macronutrients to prepare for fight week. A gradual reduction in carbohydrate intake can promote glycogen depletion and assist in weight reduction due to the water loss associated with glycogen stores [112]. Simultaneously, protein intake should be maintained to preserve muscle mass, while fat intake can be adjusted to meet caloric goals while attempting to maintain performance output [107,113]. Combining nutritional adjustments with both rapid weight loss and acute water loss strategies allows for effective weight management and readiness for fight week [37]. This tapering period and the strategies discussed will enable combat athletes to optimize their preparation for fight week and maximize their performance on fight day [22,112].

#### 5.2.3. Fight camp – nutrient timing

Combat athletes may engage in up to three training sessions per day during fight camp. As mentioned previously, training sessions may be brief (i.e. 45 minutes), or can be extended sessions (i.e. 90 to 120 min) of various intensities and metabolic demands [98]. Thus, planning meal frequency around the athlete’s training schedule aids in meeting carbohydrate and protein needs for the day. The ISSN’s Position Stand on Nutrient Timing details such strategies, and relevant points are summarized here. It is recommended athletes consume a meal two to 3 h prior to their first training session [97]. Strategies for acute fueling prior to training session (e.g. 60 minutes) include consuming high glycemic, carbohydrates (1–4 g/kg) while limiting fat and fiber intake to minimizing gastrointestinal distress during exercise. During high-intensity exercise, especially in hot conditions, athletes should consume a carbohydrate solution (6–8% concentration) to maintain blood glucose levels and hydration. Following a training session, consuming a combination of carbohydrates (≥1.2 g/kg/h) and protein (0.2–0.5 g/kg/h) post-training session enhances glycogen repletion and supports muscle recovery. Due to the multiple training sessions scheduled during fight camp, rapid refueling strategies may be adopted for sessions less than 6 h apart. The ISSN recommends a carbohydrate intake of 0.6 to 1.0 g/kg of body mass within the first 30 min after exercise, followed by carbohydrate intake every 2 h for the next 4 to 6 h to promote maximal glycogen replenishment. As mentioned previously, adding protein to carbohydrate intake enhances glycogen resynthesis. During fight camp, athletes focus on performance and longitudinal weight loss. This emphasizes the need for a well-planned diet that aligns with training schedules and fight weight goals.

#### 5.2.4. Hydration

Proper hydration is imperative for the health and performance of athletes. Even a small change of 2.5% in body weight due to dehydration can impair performance [114]. Adequate hydration should aid in supporting metabolic and training demands, reducing the risk of injury, and promoting recovery. The primary determinant of maintenance water requirement appears to be metabolic. However, total fluid requirements are highly variable and quite complex. Therefore, it is challenging to set a daily water requirement for combat athletes. Nevertheless, 1.5 ml/kcal may be suitable to cover variations in activity level, sweating, and solute load [115].

It is recommended that combat athletes focus on consuming sodium and potassium-based fluids to avoid electrolyte imbalances [98,116]. In situations where there is excessive water loss due to individual sweat rates or hot, high humidity environments, athletes may need to consume more water. For quick and complete rehydration, a simple approximation of 1.5 L of fluid per kg weight loss is recommended [111]. Fluid loss and intake must be closely monitored during a fight camp to gauge sweat rates. A good strategy to manage fluid balance is to take measurements of the athlete’s body weights preceding and following training sessions [48]. This may aid the sports nutrition professional in ensuring proper fluid replenishment and provides essential information regarding acute water loss and sweat rates that can inform weight-making tactics during fight week.

During fight camp, heat acclimation activities can be beneficial for combat athletes. The primary goal of heat acclimation is to increase the amount of sweat produced by sweat glands, thus increasing overall whole-body sweat rate [117]. Heat acclimation is induced by cumulative exposure to heat stress which in turn leads to physiological adaptations including an increase in plasma volume, increased sweat rate, increased cutaneous vasodilation and more efficient sweating over the body’s surface [118]. With habitual activation (e.g. heat acclimation), sweat glands demonstrate plasticity in their size and neural/hormonal sensitivity, which in turn impact sweat rates and sweat [Na^+^] [117]. It is of notable importance to consider that sweat rates vary amongst individuals. Generally, individuals who are heat acclimated will start to sweat earlier and will have a higher sweat rate while exercising/training. Additionally, it is commonly understood that athletes with a larger body mass and BMI will sweat more readily compared to individuals with a smaller body mass. Nevertheless, two individuals with a comparable body mass and similar exposure to heat acclimation may have differing sweat rates due to genetic distinctions, surface area-to-mass ratio, aerobic capacity, exercise intensity, and environmental conditions [119]. It should also be noted that there are seasonal adaptations in sweat rates from winter to summer, and athletes consistently living and training closer to the equatorial latitudes, particularly with high humidity, may have increased whole-body sweat rates and water loss [120]. Hence, monitoring sweat rates during fight camp can provide valuable insight into combat sport athletes’ daily fluid losses from training and/or heat acclimation activities.

Because of the limited published literature on heat acclimation and sweat rates in combat sports, the authors propose the following protocol which has been derived from methods currently adopted by combat athletes. Heat acclimation protocols can be planned during the fight camp and throughout longitudinal weight descent. Protocols should begin 4–5 weeks prior to the acute water loss phase during fight week. With respect to the proper modalities, athletes can use the sauna 3–4 times weekly for 15–25 min or hot water immersion for 20 min 2–3 times per week. Acclimation starting points and frequency of sessions will vary based on the athlete’s weight, body composition, typical fluid turnover during training, and training environment (temperature and humidity).

#### 5.2.5. Longitudinal weight descent - practical applications

- Without modalities that measure RMR, utilize the Mifflin St Jeor or Cunningham equations to determine daily energy requirements.
- Aim for 0.5–1 kg in body mass loss per week. For more aggressive weight loss, create a larger caloric deficit, but do not drop below the athlete’s RMR.
- Adjust caloric intake as needed during the weight descent proportionate to losses in body mass.
- Monitor the athlete’s rate of weight descent.
- Carbohydrate intake should not drop below 3–4 g/kg/d.
- Protein intake during weight descent should be 1.6–2.2 g/kg/d.
- Fat intake can range from 0.7–1.3 g/kg/d and may need to be lowered to create an energy deficit and drive body mass loss.
- Monitor fluid turnover rates and rehydration during training and sparring.
- Numerous factors contribute to sweat rates, such as genetics, body mass, body surface area to mass ratio, climate, humidity, and acclimation to heat.
- If necessary, heat acclimation strategies may be used to promote increased whole body sweat rates.

### 5.3. Nutritional considerations the injured athlete

Combat athletes participate in sports where the primary goal is engaging in direct contact with the opponent. Many of the combat disciplines wear minimal protective equipment and thus injuries are inevitable. Common injuries occur to skeletal muscle, bone, tendon, and ligament. Injuries normally occur to the head, specifically in the head, wrist/hand and knees and shoulders. The most frequent injury types are lacerations, fractures, and concussions [3]. Although injuries frequently occur during a competition, injuries outside of a match are also commonplace. Overtraining and overuse injuries, stemming from the demanding training schedules can also occur and contribute to the increased risk profiles of combat athletes’ [121,122].

Though several nutrition strategies exist to address injury prevention and recovery, a hierarchy is commonly followed. Inadequate dietary intake can increase the risk of injury, impair performance, and contribute to an overproduction of stress hormones [123]. Consequently, the top priority that should be addressed is ensuring energy balance is maintained as outlined in the General Preparation section. When the energy intake is insufficient, the body will start to further break down lean mass to use as energy [124]. Though protein intake plays a crucial role in protein turnover and lean mass maintenance, it is important to keep in mind that when energy intake is insufficient, protein may be required to contribute to energy demands [124]. Due to protein’s role in tissue repair and lean mass maintenance, protein requirements will therefore be even greater during injury. Intakes as high as 2.3 g/kg/day can minimize muscle loss during energy deficits in active individuals [47,125]. In critical care patients, intakes ranging from 2.0 to 2.5 g/kg/day support recovery and healing [126]. Based on the literature, the authors recommend 1.3–2.5 g/kg/day of protein to promote healing and maintain lean body mass during injury recovery. Distributing protein intake throughout the day may make it more manageable for athletes to meet their protein intake goals. It is also critical to be aware of the micronutrient needs during the period of injury. Micronutrients are necessary to support tissue repair and recovery. For example, vitamin D positively affects the immune system and combats inflammation in muscles and bones [48]. Vitamin C and zinc are important for the immune system and are frequently used in wound healing [48]. Other dietary supplements can also be considered for their potential effects in promoting recovery (e.g. creatine monohydrate, fish oils, curcumin, probiotics, and amino acids [Table 3]).

**Table 3.** Dietary Supplement Considerations for Combat Athletes.

Supplement	Rationale	Recommendation
Multivitamin	Help athlete meet daily micronutrient needs and minimize risk of deficiencies	Daily supplementation
Vitamin D (calciferol)	Low vitamin D status increases risk for injury and upper respiratory infections [206]	1000 IU/day [48]
Vitamin C*	Enhances iron absorption and supports immunity. *Serves as an antioxidant and aids in oxidative stress which may help recovery post TBI	Males 90 mg/d Females 75 mg/d [86]
Iron	Athletes unable to maintain sufficient iron levels, possibly due to low intake.	18 mg/day for women and >8 mg/day for men [48]
Magnesium	Evidence supports use of supplementation when deficiency present.	Current RDA is Males 420 mg/d and Females 320/d, as high as 500 mg/d [86]
Zinc	Supports immune function	Males 11 mg/d Females 8 mg/d86
Omega-3	Supports recovery, anti-inflammatory properties. Supports brain health and function	>2 g of EPA and DHA daily [207]
Creatine	Enhance acute exercise capacity, increase muscle creatine stores and increase lean body mass. Offers neuro protective effects for post TBI management	3–5 g/day [86]
Caffeine	Enhance exercise performance, delay onset of fatigue	3–6 mg/kg body mass [208]
Sodium Bicarbonate	Supports high-intensity exercise	0.2 to 0.5 g/kg [209]
Beta alanine	Enhances exercise performance via increases in intracellular buffering capacity	4 to 6 g of beta-alanine for at least 2 to 4 weeks [64]
β-Hydroxy β-methylbutyrate	Supports lean body mass	1–3 g or 38–40 mg/kg/bw daily [94], [95]
Antioxidants	Support brain function, reduce inflammation and protective effects from oxidative damage	Found in fruits, vegetables, curcumin, N-Acetyl-Cysteine, Vitamin E, Glutathione, Coenzyme Q_10_ B-vitamins [138]

#### 5.3.1. Nutrition for traumatic brain injury

As with other impact sports, the prevalence of traumatic brain injuries (TBIs) in combat sports is high, particularly during fight camp when sparring and live drills are often emphasized. The causes of TBI in combat sports vary from direct impact or hits to the head, knockouts, or takedowns involving blunt force to the head [127]. TBIs range in severity and are classified as mild, moderate, or severe. Many fighters have experienced some form of TBI during their careers [127,128]. Thus, nutrition strategies implemented following TBI may aid in their recovery and timely return to sport [129,130].

Following a TBI, nutritional considerations are aimed at supporting repair and recovery, reducing inflammation and oxidative stress, neurogenesis, and restoring function [131]. As with general injury nutritional management, adequate protein and energy are paramount. Research suggests that increasing protein and energy intake in the hours following a TBI improves neurological outcomes and lowers mortality rates [132,133]. During TBI recovery, energy needs are estimated to increase between 100% and 200% of baseline resting energy expenditure values [134]. Equations to predict resting energy expenditure (Mifflin St-Jeor of Cunningham) or indirect calorimeters should be used to estimate baseline resting energy expenditure. Protein intake of approximately 2 g/kg/d total protein further supports recovery from TBI [135]. Supplementation can also aid the recovery process as well as help combat athletes consume the therapeutic doses of essential nutrients. Research suggests that creatine may have neuroprotective effects [136] by minimizing inflammation and preserving brain tissue [136]. Studies in adolescents reported improvements in cognitive function and reduced headaches, dizziness, and fatigue with creatine supplementation following mild TBI [137]. Other supplements including omega-3 fatty acids, antioxidant supplements, and B-vitamins also support brain function and are shown to reduce the severity of brain injuries [129,130]. Omega-3 fatty acids, commonly found in fish oil supplements, have anti-inflammatory properties, and may help reduce neuroinflammation [131]. Antioxidants such as coenzyme Q10, vitamin C, B vitamins and vitamin E are thought to mitigate oxidative damage following TBI [138]. N-acetylcysteine (NAC) is a precursor to glutathione, and glutathione offers neuroprotective effects from oxidative damage stemming from brain injuries [139]. Although such clinical nutritional therapies do not cure TBIs, there is compelling evidence supporting nutrition being beneficial in promoting recovery from brain injuries. Furthermore, inadequate nutritional intake impairs the recovery process.

### 5.4 Fight week/rapid weight-loss strategies

Weight cutting is a process that includes weight loss (RWL) to qualify for a desired weight class division, followed by rapid weight gain (RWG) to recover body mass losses before the start of the competition [36]. When official weigh-in occurs on the day before in MMA, approximately 24–36 h remain prior to the beginning of competition. This duration is thought to be an adequate amount of time for athletes to rehydrate and refuel sufficiently and safely [140].

Weight can be lost rapidly through, water loading, fluid restriction, acute dehydration, hot water immersion/salt baths and sauna, fasting, and reduced carbohydrate and fiber intakes [141,142]. Barley et al. provided insights into the weight cutting strategies across combat sports disciplines. Six hundred thirty-seven athletes from judo, Brazilian Jiu Jitsu, boxing, MMA, and Muay Thai/kickboxing were surveyed online. Results showed that 87% of combat athletes included food restriction (skipping meals, reducing overall energy intake to <22 kcal/kg), 83% used water loading (hyperhydration), 79% restricted fluids (hypohydration), 51% used sauna, 85% increased training, and 43% used sweat suits and plastics [138,143]. Additionally, some combat athletes may use potentially harmful methods, such as diuretics and laxatives to lose excessive body weight as they aim to compete in the lowest possible weight class [99]. Such practices have a detrimental effect on health, well-being, mood, and performance [107,144].

There have been studies that have reported weight cutting practices to have no negative effect on exercise performance [37,145]. However, there remains a limited body of scientific research investigating rapid weight-loss strategies, which includes evaluations of the different methods, the influence on competitive performance, acute physiological changes, and refueling and recovery methods to improve performance following rapid weight loss [99]. The existing literature on the current practices for several rapid weight-loss strategies is discussed in this section.

#### 5.4.1. Glycogen depletion

Dietary carbohydrates are stored in skeletal muscle (350–700 g) and liver tissue (80–100 g) as glycogen and act as readily available energy reserves for glucose metabolism with final concentrations being dependent upon training status, diet, muscle fiber-type composition, sex, and body mass [146]. Glycogen is a branched polymer of glucose that binds to water at a ratio of 1 g glycogen to 2.7 g water [147]. Glycogen can contribute as much as 1–2% of skeletal muscle mass and up to 8% of liver mass [112,148]. In consideration of this, previous research has shown that 7 days of a low carbohydrate diet, combined with training and a slight reduction in energy intake (<10%), can achieve a body mass reduction of 2% while maintaining strength/power measured via a 30-s Wingate test [149]. A similar body mass loss was achieved following a 14-day period of carbohydrate restriction. Thus, due to the known association of water content with endogenous glycogen content, glycogen depletion continues to be a common means for combat athletes to reduce body mass.

A low carbohydrate diet can aid in glycogen depletion. Although combat athletes need carbohydrates for performance, adhering to <50 g carbohydrates/day during the rapid weight-loss phase prevents glycogen replenishment. The need for carbohydrates to drive high-intensity work, and its connection to body mass via glycogen status create an ongoing conflict for the combat athlete. The extent to which glycogen levels can be manipulated is primarily influenced by carbohydrate intake and the existing glycogen status before implementing glycogen-depleting strategies [150]. The rate/amount of glycogen depletion is in part predicated on the athlete’s typical carbohydrate intake, which should be calculated in grams and calories. In addition, the degree to which one can/should manipulate glycogen status is limited by the recovery window (i.e. time from official weigh-in to competition) of different combat sport disciplines and the regulations of the promotion. Care should be taken to evaluate the cost/benefit of additional exercise in the period just prior to weigh-ins versus a longer period of carbohydrate restriction prior to competition [151]. Ultimately, exercise intensity, duration, and carbohydrate feeding will dictate the rate of glycogen depletion. During fight week, training sessions typically include technique work, drills, and additional aerobic exercise, often under the auspices of the skill coach. Many skills coaches have combat athletes continue technique work and drills during fight week, which may result in greater skeletal muscle glycogen depletion due to the high-intensity nature of the activity. If the athlete is restricting dietary carbohydrates and continues high-intensity skills training, glycogen stores can be significantly depleted within hours.

Therefore, training intensity and frequencies, carbohydrate intake, and body weight should be carefully monitored to ensure an effective weight cut. Accordingly, because total calories, carbohydrates, and water may be restricted, if additional activity is needed for glycogen depletion and rapid weight loss, low-intensity steady state (LISS) cardio may have utility during fight week [151]. To achieve this, an exercise intensity ranging between 40% and 50% of maximum heart rate for 30–45 min should be suitable as an additional method for rapid weight loss. Consequently, the sports nutrition professional must work in concert with the fight week strategies of the skills coach and monitor training frequencies and intensity, exercise protocols, and losses in body mass, to perform glycogen depletion safely and effectively.

##### 5.4.1.1. Glycogen depletion practical – applications

- Before glycogen depletion is initiated, the sports nutrition professional should calculate the combat sport athlete’s average daily carbohydrate intake.
- Glycogen depletion can begin as early as seven days prior to weigh-in. For most combat athletes, a minimum of 72 h is sufficient.
- Following a low carbohydrate diet (2–3 g/kg/d) may be required to prevent the restoration of muscle glycogen stores following their depletion via skills training and/or additional cardiovascular activity.
- Plan carbohydrate intake and glycogen depletion in accordance with fight week training intensities and volumes.
- Glycogen depletion can result in a loss of 1–2% of body mass.
- The total amount of carbohydrate restriction during fight week will also be subject to how much body mass must be lost for the combat athlete to achieve their desired weight.
- Carbohydrate restriction as a practice should be limited when possible and implemented only as needed for rapid weight loss.

#### 5.4.2. Fiber manipulation

The use of bowel preparation formulas, such as those used in clinical situations to prepare the gastrointestinal tract for surgery (i.e. laxatives), is not uncommon among weight class athletes. Indeed, such tactics can be used to facilitate the removal of intestinal contents and promote acute water loss [152]. While these methods may be effective for expelling the colon of intestinal bulk, the use of clinical bowel preparation formulas (e.g. osmotic laxatives and purgatives) has been shown to reduce exercise capacity and ultimately overall performance and therefore it is not recommended [153]. Instead, dietary strategies to reduce total food volume may therefore be a preferable way to manipulate the mass of intestinal contents while maintaining performance [152].

Dietary fiber can slow the transit time of food through the bowel and draw water into the intestinal space, thereby adding bulk to stools [152]. Different foods possess different fecal bulking properties [154], and ultimately the changes in habitual fiber intake, particularly non-fermentable fibers, can elicit changes in body mass. Estimated fermentability has been shown to determine the role of fiber in total fecal wet weight. Less fermentable dietary fibers from cereals, such as wheat bran, contribute the most to total fecal weight compared to more fermentable dietary fibers, such as those originating from fruits [154]. This additional fecal matter is likely due to non-fermentable fiber’s higher water-binding capacity, as well as its greater resistance to fermentation from colonic bacteria. There appears to be a linear relationship between fiber intake and bowel cleanliness in pre-colonoscopy patients [155], and the utilization of a low-fiber diet for 2 days prior to treatment reduced bowel cleanliness compared to high-fiber diets [155]. Indeed, when a low-fiber diet (<10 g/d) was continued for 7 days, it was as effective as a bowel preparation formula [156]. In a study of 19 healthy males consuming a habitual high-fiber diet of 30 g fiber/day for 7 days followed by four of a low-fiber diet (<10 g fiber/d) that was matched for energy and macronutrients, a significant reduction in body mass was shown after 4 days of a low-fiber diet (Δ = 0.4 ± 0.91% *p* < 0.05) and on day 5 (Δ = 0.74 ± 0.99% *p* < 0.01). Subjects did report increased hunger and a decline in stool frequency; however, the diet modification was reported to be tolerated and repeatable [157].

Despite limited research on the bowel-emptying effects of a low-fiber diet, the use of low fiber diet appears to be a useful tool for reducing body mass without the need for bowel preparation formulas, which can ultimately pose a risk of undue physiological stress on the gastrointestinal system [156]. Additionally, following a low-fiber diet for a minimum of 3 days seems to be effective for rapid weight loss with minimal disruption, despite moderate increases in appetite, reductions in bowel movements, and hardening of stools. Considerations should be made to identify habitual dietary fiber intake and fiber type to better estimate and proactively account for changes in body mass when following a low-fiber diet. Furthermore, since females and older populations have reported slower GI motility, further research needs to be conducted to understand the interplay between gender and age in the effects of low fiber on body mass changes [158]. Further research is needed to establish precise guidelines for the use of low-fiber diet due to the large variations in whole gut transit times, which can range from 10 to 96 h [159]. Continued research on the implications of low-fiber diets in weight-making sports, as well as health and performance outcomes, is warranted.

##### 5.4.2.1 Fiber manipulation practical – applications

- Low-fiber diet may be used as a tool to aid RWL in weight class sports.
- Reducing dietary fiber from habitual intake (>30 g/d) to a Low fiber diet (<10 g/d) for 4 days may result in 1–2% decrease in body mass.
- Non-fermentable fibers from cereals, wheat bran, and vegetables contribute to fecal wet mass more than fermentable fibers found in fruits.
- Considerations should be made for female and older athlete populations who may experience slower GI motility and bowel clearance times.

#### 5.4.3. Sodium manipulation

As combat athletes lose water and electrolytes through thermoregulatory sweating during heat exposure, aerobic activity, and skills training during fight week, it is well established that the rate and composition of sweat can vary significantly within and between individuals [160]. Scientists and practitioners may conduct sweat tests to determine sweat and electrolyte losses, particularly sodium. However, the current methodological practices and challenging field conditions often lead to inconsistent and inaccurate results. While techniques like whole body washdown, which is the reference standard, require a laboratory, more practical methods such as sweat patches have shown inconsistency and limited effectiveness [160].

Accordingly, determining how much sodium to ingest or restrict during fight week proposes numerous challenges. To establish a measure of sodium restriction, it is advisable that the sports nutrition professional first ascertain a baseline of sodium intake during fight camp and then propose a lower sodium intake of < 2.3 g daily during fight week, if needed. Due to increases in sweat loss, athletes should not consume less than the current RDA of 2300 mg/day in order to minimize sodium losses [161,162]. Thus, for athletes whose base intake is below 2300 mg, more emphasis can be put on other water manipulation modalities. Physiologically, the human body tightly regulates the osmotic pressure of body fluids through renal excretion and retention of electrolytes and fluids. A summary of literature examining the relationship between sodium intake and water intake found that in a steady state, urine volume remains unchanged over a broad range of sodium intakes. Rather, the adaptation to higher sodium excretion lies only in changes in urinary sodium concentration [163].

Water retention is responsible for the well-documented increase in body mass following acute increases in sodium intake [163]. The resulting increase in fluid intake due to thirst in conjunction with no significant changes in urine volume causes the extra fluid to remain in the body to limit the rise in plasma sodium concentration by diluting the sodium that cannot be excreted quicky enough [163]. In a steady state, if sodium levels remain within a reasonable range of (50–250 mmol/day), most of the observed adaptation will rest on changes in the concentration of sodium in the urine without any changes in urine flow rate. However, when an abrupt change in sodium occurs (e.g. 70 mmol/d to 250 mmol/d), assuming usual daily activities remain constant and without any limitations in water intake, a return to sodium and body mass balance requires 2–3 days [164].

The complexity of sodium steady state dynamics and its relationship to body mass changes is further complicated by the varying tactics utilized by combat athletes such as hyperhydration (water loading), heat acclimation, caloric restriction, diuretics, etc., during the weight-cutting period [116,143,165,166]. Despite the limited body of research on varying sodium levels and their effects on acute water loss, sodium restriction has become a common practice among some combat athletes during the weight cutting period [98]. Due to the limitations in previous research aimed at investigating the role of sodium restriction on acute water loss in combat athletes, the precise degree to which sodium needs to be reduced is unknown. Further research is warranted.

##### 5.4.3.1 Sodium restriction – practical applications

- Sodium restriction should be based on athlete’s usual intake and anticipated/needed water loss.
- Sodium restriction recommendations may vary greatly, and there is a paucity of research associated with restriction and acute water loss. A low sodium diet of < 2.3 grams of sodium during fight week may be suitable.
- Less than 2.3 grams of sodium per day is deemed low for the general population, individuals with significantly lower rates of sweat loss and sodium loss than combat athletes, hence caution should be taken in dropping levels significantly below 2.3 grams.
- Monitoring both weight and fluid loss in relation to sodium restriction will determine if sodium restriction is suitable or if additional restrictions are needed.

#### 5.4.4. Polyuria/water manipulation

Urine excretion is tightly regulated by the renal system. Fluid balance is achieved by aldosterone and anti-diuretic hormone triggering a renal response to either release or conserve fluid thereby maintaining total body water as well as electrolyte balance [167]. Urine excretion is the body’s primary fluid balance regulator and as such, typical urine losses range from 1–2 liters per day [168,169]. Urine volume decreases with dehydration, with the obligatory rate of urine production to excrete metabolic waste being 0.5 liters per day [167,170]. The upper limit for urine production is 1.8 liters per day; therefore, fluid intake that exceeds urine production limits may lead to hyponatremia [171].

To encourage polyuria and thus acute water loss, the use of pharmacological diuretics to promote greater urine loss has been reported among combat athletes [166]. In a survey of 637 combat athletes, the prevalence of reported diuretic use was 12% overall, with the lowest reported use in judo of 5% and highest of 12% in Tae Kwando [143]. The survey did not specify the type of diuretic used which may include pharmaceutical grade diuretics and/or over-the-counter products such as caffeine anhydrous. The lower prevalence of diuretic use may in part be due to the increase in testing and regulatory bodies that monitor athletes given that pharmacological diuretics are included in the World Anti-Doping Agency’s List of Prohibited Substances [172].

A commonly adopted method for inducing polyuria amongst combat athletes is water loading or hyperhydration. Previously, water-loading practices in combat sports had been largely based on anecdotal evidence. Athletes and sports nutrition professionals prescribed varied recommendations for total fluid consumption and the durations in which water loading should be applied. To date, there is not a robust body of research on the subject. Water loading is a strategy characterized by the consumption of large volumes of fluids for several days prior to fluid restriction. A rapid weight-loss questionnaire of 314 mmA athletes showed that the frequency of water loading occurred “always” or “sometimes” in 72.9% of the athletes surveyed [173]. Barley et al. examined acute water loss habits in 637 combat athletes and revealed a water-loading prevalence of over 60% in MMA, Muay Thai, kickboxing, and boxing, with an overall prevalence of 53% among all combat sports [143].

A study which provided significant insight on the efficacy and safety of water loading was conducted by Reid et al. [109]. They investigated the effect of water loading for 3 days (100 ml/kg/d) compared to a control group (40 ml/kg/d), followed by fluid restriction on day 4 (15 ml/kg/d). Significant body mass loss was observed in the water-loading group on day 5, with a loss of 3.2% compared to 2.4% in the control (CON) group [116]. The difference in body mass loss in the water-loading group was attributed to greater fluid loss relative to fluid intake during the period of fluid restriction, compared to the control group. Despite concerns regarding hyponatremia, the study showed no evidence of problematic blood chemistry changes or impaired physical performance following rehydration. Moreover, a 3.2% loss in body mass represents nearly half of the 6.7% above scale weight at 72 h prior to weigh-ins.

Another study conducted by Cho & Han [174] included 13 university wrestlers (weight 71.5 ± 8.0 kg, BMI 25.0 ± 2.0 kg/m^2^) randomly divided into the weight loss (WL) group (*n* = 6) and water-loading weight loss (WWL) group (*n* = 7). Wrestlers performed a two-week weight-loss program, targeting an average of 5–10% of body weight reduction, under the supervision of a coach. Participants were instructed to drink 1.5–2 liters and 6–7 liters in the weight loss and water loading weight loss groups, respectively, for two weeks prior to weigh-in. Neither group consumed water the day before weigh-ins. Anthropometric characteristics, hematocrit (HCT), serum electrolytes, aldosterone, and cortisol were measured before and after weight loss. Results showed the weight-loss and water-loading weight-loss groups showed a decrease in body mass of 8.2% and 11%, respectively. These numbers supersede the 2.4% and 3.2% loss in body mass observed by Reid et al. [109]. However, it must be considered that the wrestlers underwent approximately eleven additional days of water loading, caloric deficits, and intense training. Hence, the total loss in body mass of 8.2% and 11% also includes changes in body composition. Of notable importance, there were no negative implications on blood chemistry and the researchers concluded that large-volume hydration before water restriction for short-term loss in body mass can be completed safely under the parameters with which this study was conducted.

#### 5.4.5. Acute water loss

The manipulation of body mass via sweating is the most common method of rapid weight loss undertaken by combat athletes [152]. Although sweat losses are commonly used, there are currently no generalized standards to determine the ideal method, the optimal duration of exposure, and the most suitable environmental factors. However, acute water loss strategies must be applied while minimizing overheating and adverse performance and health outcomes.

Despite previous research suggesting that combat athletes lose nearly 10% of their total body mass pre-weigh-in [107,141,165,166,175,176], solely utilizing sweat loss to achieve a 10% decline in body mass within 24 h of weigh-in is ill-advised and potentially dangerous to health. With several factors notwithstanding, body mass losses ranging from 5% to 8% can have varying degrees of impact on health and performance. Some data indicate that certain athletes can achieve this decline in body mass via acute water loss and not compromise performance and health [166]. Nevertheless, to date, this remains inconclusive.

A major challenge is understanding which acute water loss methods should be utilized to achieve the decrease in body mass leading to weigh-ins; and what is the true starting point to initiate acute water loss tactics. The starting point prior to acute water loss should represent body mass in a euhydrated and well-nourished state (i.e. prior to glycogen and fiber depletion) to take advantage of non-essential compartments of bound water.

In a research study that captured weight loss data in 600+ successful UFC athletes’ weight cuts, it was shown that there was a significant reduction in non-essential body mass in the 24–72 h window prior to weigh-ins. UFC fighters were approximately 6.7% above their weight class 72 h before weigh-ins, but were only 4.4% over their weight classes 24 h prior to weigh-ins [177]. Although the hydration status in the 24-h window before weigh-ins was not published, a 4.4% body mass loss represents a much smaller and therefore feasible amount of acute water loss.

It has been noted that when women and men were matched for surface area-to-mass ratio and fitness level, both had similar heart rate and core temperature responses to heat acclimation. Despite this, sweating responses were greater in men, and they required a shorter heat acclimation period (4–5 days) compared to women [118]. With the growing number of female participants in combat sports, it is important to consider key differences in sweat rate and heat tolerance. Female thermoregulatory responses vary throughout the menstrual cycle due to the influences of reproductive hormones estradiol and progesterone. During the luteal phase or high hormone phase of oral contraceptive use, thermoregulatory control of both sweating and cutaneous vasodilation is shifted by approximately 0.5 Celsius to higher core body temperatures [118]. In hot and dry conditions, males experience a smaller change in core temperature compared to women, whereas the opposite is true in hot and humid climates. Men have a higher overall sweating capacity, which allows them to respond more efficiently to dry heat [118]. It is worth noting that women have a lower sweat capacity than men for a given amount of metabolic heat generation, and they are also less responsive to the effects of training adaptation on sweat rate and sweat temperature threshold [118]. Women between 18 and 35 years of age are also five times more likely to experience symptoms associated with orthostatic intolerance compared to men of the same age and health status [118]. Despite these differences in sweat rate and adaptations, the role of sex hormones in thermoregulation and acute water loss (adaptive whole-body heat loss) is complex, and further research is needed.

The prevalence of sweat loss strategies for acute water loss is not surprising considering the relatively large body mass losses that can be achieved in a rapid and time-efficient manner, with sweat rates up to two liters per hour having been recorded [168]. The onset of sweating can be altered by changes in plasma electrolyte concentration, plasma volume, and total body water content. However, these factors are not optimized to enhance sweat losses without the introduction of more fluid into the body, which is contrary to the end goal of acute water loss [152].

Variables that may dictate the rates and volume of acute water loss includes:

- Environment: increased ambient heat increases core temperature and increases the need for sweat to be produced to dissipate heat [178]
- The athlete’s heat acclimation preparation
- Clothing: insulating clothing traps heat thereby increasing core temperature and sweat rate and dark colors can increase the amount of radiant heat an athlete is exposed [178].
- Duration and intensity of physical activity: higher exercise intensity (higher percentage of VO_2_Max) requires increased energy and therefore respiration which in turn generates more heat. The increased heat production leads to an increased sweat rate to compensate and cool the body [178].
- Individual physiology: body’s surface area, heat acclimation and genetic/sex differences can cause variations in sweat rate [178]
- The athlete’s acute water loss strategy preferences and psychological state

Generally, acute water loss strategies can be classified into two categories: active sweating (exercise or skill training often with the aid of a sauna suit or layered clothing for insulation) or passive sweating (exposure to hot environment, e.g. sauna, steam rooms, infra-red saunas, wraps and/or hot baths). It is important to note the differences in physiological response to active versus passive sweating. Cutaneous vasodilation and sweating during heat stress results in a redistribution of blood flow to the periphery and a requirement for increased cardiac output to meet the thermoregulatory demand. Passive sweating prior to active sweating decreases plasma volume, sweat rate, and body heat storage [179]. These physiological changes occur to a lesser extent when hypohydration develops only during exercise [179]. When combat athletes are in a very depleted and hypo-hydrated state, they may need to limit active sweat loss methods. During exercise, the mismatch between venous return and limited cardiac output is mitigated by the muscle pump which propels blood back to the heart. The lack of muscle pump or sudden cessation of exercise in addition to increased peripheral venous compliance can result in a transient decrease in cerebral perfusion pressure causing orthostatic intolerance, dizziness, fatigue, and syncope [118].

Traditionally, many combat athletes make use of combining training sessions (active) with fluid restriction to promote dehydration. Active sessions are often used with additional passive methods for acute water loss. Tangible arguments for, and against, active strategies can be made. Active strategies such as high-intensity skills training, shadow boxing and striking pads require increased energy expenditure and oxygen demand which in turn generates more heat, leading to an increase in sweat rate to compensate and cool the body. This can result in the athlete accumulating considerable acute water losses. Additionally, many skills coaches practice active methods, such as striking and technical sparring, with the notion that this maintains the motor skill applications which were advanced during the general preparation and fight camp periods. Moreover, the increased loss of peripheral skeletal muscle glycogen associated with active methods such as hitting pads and striking, may further contribute to water loss. If the combat athlete has greater than the average of 4.4% body mass to lose within 24 h of the official weigh-in, both active and passive strategies may be warranted in some situations. Nonetheless, as discussed, reduced plasma volumes from acute water loss can alter changes in cardiac output, flow, distribution, and blood pressure, which in turn can impair the efficiency and safety of active methods such as skills training and technical sparring [118].

It is of notable importance that many coaches, fighters and teams have established methods for acute water loss and the sports nutrition professional may have to operate within the preferred parameters of these strategies. As an example, if the team uses active methods and several skill application sessions as part of acute water loss methods, then passive strategies should be used only when required and when ample time is available for recovery. As with all nutritional strategies in combat sports, particularly as it pertains to acute water loss requirements, the sports nutrition professional is often required to evaluate what is optimal and what is necessary.

Passive strategies may allow the combat athlete to spare muscle glycogen and reduce the elevated physiological demands which active strategies may induce on both the nervous systems and skeletal muscles. The frequency and duration of passive strategies will be contingent on how much body mass must be reduced within 24 h of weigh-in. Passive strategies, as with all acute water loss methods, may be appropriate when sufficient post-weigh-in recovery time is available. Nevertheless, passive strategies must be strategically applied to optimize the volume and rate of acute water loss [166]. It is also prudent that the sports nutrition professional ensures that combat athletes do not overheat from passive strategies for acute water loss. Monitoring the athletes core temperature is a method to avoid overheating, heat stress injuries or potential hyperthermia. The core temperature in which such conditions may occur is not exact and highly variable between individuals. However, hyperthermia is defined as a body temperature greater than 40° Celsius (104° Fahrenheit) [180]. Therefore, independent of the cumulative exposure to heat acclimation the combat athlete has undertaken, it is imperative to avoid heating near this core temperature. As with all acute water loss strategies, the pros and cons are contingent upon any contraindications, the coexisting practices, and the demands of the combat athlete.

#### 5.4.6. Passive weight cut strategies

In general, passive weight cut strategies refer to those modalities in which combat athletes undergo exposure to heat for acute water loss. These strategies are most often done without extensive physical activity.

##### 5.4.6.1. Hot baths

Hot water immersion is a common, passive method, used by combat athletes to induce acute water loss. A survey of rapid weight-loss practices by MMA athletes indicated that 76% of fighters report using hot baths either “always” or “sometimes” [141]. Hot water immersion has also been coupled with the addition of various salts, often Epsom salts, to further enhance acute water loss. Connor and Egan [181] investigated the magnitude of body mass losses during hot water immersion with or without the addition of salt, with the temperature commencing at 37.8°C and being self-adjusted by participants to their maximum tolerable temperature. In a crossover design, eight male MMA athletes (29.4 ± 5.3 y; 1.83 ± 0.05 m; 85.0 ± 4.9 kg) performed a 20-min whole-body immersion (39.0–39.5°C) followed by a 40-min full body wrap in a warm room (24.0–29.0°C), twice in sequence per visit. The full body wrap included a knitted wool hat, cotton t-shirt, hooded cotton sweatshirt, cotton tracksuit bottoms/sweatpants, and socks. Participants were then covered in blankets on a bed in an adjacent bedroom with only their faces exposed.

Additionally, the athletes restricted carbohydrates, fiber, and fluids as commonly practiced during fight week. Body mass losses induced by just the hot bath protocols were 1.71 ± 0.70 kg and 1.66 ± 0.78 kg, for the freshwater bath and saltwater bath, respectively. Hence, demonstrating no further loss from saltwater baths [181]. The total loss in body mass from hot water immersion was equivalent to 2.0% of body mass. Additional losses were shown from 40-min wraps in a warm room. The combined protocols of hot water immersion and full-body wraps produced a 4.5 ± 0.7% decrease in body mass. In conclusion, hot water immersion can be an effective passive technique for acute water loss with added salt, creating no further losses of body mass.

##### 5.4.6.2. Hot water immersion – practical applications

- Hot water immersion is an effective method that can be applied within 24 h of weigh-making activities (i.e. weigh-ins) with its effectiveness being contingent upon the loss in body mass required and whether other rapid weight loss and acute water loss modalities are being applied.
- Water temperature typically ranges from 39–40°C (102–104°F) or potentially higher if tolerated by the athlete for durations ranging from 15–20 minutes.
- Ambient room temperature, air current and humidity levels are often controlled.
- Though Epsom salt is sometimes added to baths, recent studies have shown no increase in loss of body mass from their addition.
- Carefully monitor the athlete’s core temperature during hot water immersion to prevent hyperthermia (~40°C, ~104°F).

###### 5.4.6.3. Dry saunas

There is a paucity of data which has directly investigated the effects of dry sauna exposure on rapid weight loss in combat athletes. Brito et al. surveyed 580 combat athletes participating in grappling (judo, jujitsu) and striking (karate and Tae kwon do) combat sports in the state of Minas Gerais, Brazil. They found 50% of athletes use saunas as a method of rapid weight loss [182]. Challenges remain with studies that have attempted to quantify either absolute water loss and/or water loss expressed as a percentage of initial body weight, in both women and men.

Numerous factors can play a role in the amount of water loss due to sauna exposure such as body mass, body surface area, body composition, biological sex, hydration status, physical activity level, frequency of sauna use, acute hormonal influence (e.g. vasopressin, aldosterone, etc.) ambient temperature and humidity of the sauna, and the length of time in the sauna. Additionally, some research measures water loss due to saunas using a traditional scale, while other research uses bioelectrical impedance analysis (BIA). These differences in methodology may make comparisons between different studies difficult to interpret. Podstawski et al. [183], examined the effect of dry sauna on acute weight loss in men using calibrated body mass scales. The participants were repeatedly exposed to short sauna baths [4 × 10 min; 5-min breaks between sauna sessions; temperature of ~ 90°C (~194°F) at 14–16% relative humidity] [183]. This specific sauna protocol led to a significant loss of 0.65 kg of body mass through sweat loss [183].

Another study measured acute water loss in 674 young men and women (19–20 y) exposed to two different 10-min dry sauna sessions [~90°C (~194°F) at 35% relative humidity, separated by 5 min (which included 30 s of cooling in 10°C water)]. The investigators reported decreases in body mass measures via a calibrated scale between 0.25 and 0.7 kg and 0.32–0.82 kg in women and men, respectively [184]. Interestingly, as a percentage of overall body mass lost via sweating, both females and males lost between ~0.5–0.9% of their initial body mass [184]. It should be noted that the more overweight or obese the participants were, the greater amount of water loss was observed [184]. Conversely, the least amount of water loss was recorded in the underweight subjects in both sexes [184]. Similar water losses when expressed as a percent of body mass lost (~0.9%), following a sauna bath, have also been reported in earlier research [185].

###### 5.4.6.4. Dry sauna – practical applications

- Dry saunas utilize a heating element to heat ambient air which in turn raises body temperature via conduction and convection of heated air in addition to radiation from the heated surfaces in the sauna room.
- Dry sauna temperatures can range from 60–90 °C (140–192°F).
- Due to the significantly higher ambient temperature, care must be taken to minimize the chances of overheating while utilizing the sauna. Cooling strategies such as icing of the head, neck and palms can be implemented.
- Duration of dry sauna heat exposure is highly individualized with exposures lasting from as little as 5 minutes to as long as 20 minutes.
- Research shows average fluid losses of 0.27–0.68 kg and 0.32–0.82 kg in women and men, respectively, which is equivalent to 0.5–0.9% loss in body mass.
- Numerous factors will affect sweat rates during fight week, including body mass, body surface area, body composition, biological sex, hydration status, physical activity level, frequency of sauna use, acute hormonal influence (e.g. vasopressin, aldosterone, etc.) ambient temperature and humidity of the sauna, and the length of time in the sauna.
- Sports nutrition professionals must be prudent when deciding if dry sauna should be part of an acute water loss protocol and the duration and temperature of exposure which is needed.
- Carefully monitor the athletes core temperature during any type of sauna use to prevent hyperthermia 40°C and 104°F.

###### 5.4.6.5. Steam rooms

Research identifying precise rates of acute sweat loss that can be achieved through exposure to wet saunas or steam rooms is also scant. Pilch et al. ^186^investigated the differences in acute weight loss and subjective feelings of thermal comfort between wet sauna (~59°C/138°F and 60.5% humidity) and a dry sauna (~91°C/196°F and 18% humidity) in young (22-24y) females. The sauna protocol (for both wet and dry sauna) consisted of three separate 15-min intervals separated by 5-min rest/cooling intervals. Overall, the average weight loss was significantly greater in the dry sauna (~0.37 kg) compared to the wet sauna (~0.25 kg). Additionally, rectal temperature, heart rate, and subjective feelings of thermal discomfort were greater in the wet sauna [186]. The same pattern of acute weight loss (dry sauna vs. wet sauna was 0.72 kg and 0.36 kg, respectively) were also shown in men subjected to the same basic study design (three separate 15-min intervals separated by 5-min rest/cooling intervals) using calibrated scales [187]. The research suggests that dry sauna may be a more effective and comfortable protocol for combat athletes during acute water loss sessions [186]. Some of the reasons include:

- Steam rooms utilize steam from evaporating water to heat ambient air.
- Steam rooms can have humidity levels at 95–100% and typically have lower ambient temperatures (50 °C, 122 °F)
- To prevent scalding, steam rooms often operate for short intervals making the management of ambient temperature and humidity challenging during acute water loss.
- Tracking sweat rate can also be challenging due to the high humidity and condensation of water on the skin’s surface. When sweating does occur, due to the high humidity level the body’s natural evaporative cooling mechanisms are rendered ineffective, increasing the risk of overheating.
- Dry sauna in the limited literature demonstrates higher sweat rates than steam and may be a more effective and comfortable option.
- Due to these challenges, steam rooms should be avoided, if possible, for acute water loss in favor of more controllable modalities.
- Carefully monitor the athletes core temperature during any type of steam room use to prevent hyperthermia 40°C and 104°F.

###### 5.4.6.6. Infrared saunas/portable saunas/sauna blankets

As with the dry sauna and steam room methods, there is limited study on the efficacy of infrared saunas. Conventional saunas use heating elements to raise air temperatures within the units. Infrared saunas use infrared light to heat your body while the air temperature remains relatively unchanged. Accordingly, a combat athlete’s core temperature can rise without having to sit in a room with ambient temperatures close to 200°F. Logically, this may be a viable option for combat sport athletes. Infrared saunas, portable saunas, and sauna blankets utilize infrared radiation to heat the body’s surface.

- Temperature ranges vary by model. In some models, temperatures can range from 35°C (95°F) on the lowest setting to 70 °C (160 °F) on the highest setting.
- Portable saunas and sauna blankets allow the athlete’s head to be exposed to ambient air thereby presenting greater opportunities for sweat rate tracking and implementing cooling strategies such as icing.
- Portable saunas/sauna blankets are easy to transport and have great utility, hence their rapid adoption as an acute water loss modality.
- Carefully monitor the athlete’s core temperature during any type of sauna use to prevent hyperthermia 40°C and 104°F.

###### 5.4.6.7. Mummy/burrito

Mummy wraps are a commonly utilized modality that entails wrapping an athlete (apart from the head) in warm towels or a combination of thermal mylar blankets with towels. Some combat athletes may or may not choose this approach. Mummy wraps are often used in unison with other acute water loss techniques. Scientific researchers have yet to thoroughly investigate this strategy and its efficacy, particularly as an isolated strategy. Nevertheless, the aforementioned study by Conor and Eagan [181] used wraps after two sequences of 20 min of hot water immersion. The athletes wrapped in heavy clothing and blankets in a room (24–29 C) for 40 min after 20 min of hot water immersion. The process of wrapping post hot water immersion did lead to additional losses in body mass of 2.79 kg more than hot water immersion alone. Combined, 20 min of hot water immersion and 40 min of wrap for a total of 60 min were performed and then repeated. The athletes lost a total of 4.5 ± 0.7% of total body mass. Therefore, this combination may be an effective approach for acute water loss ranging from 14 to 16 h before weigh-in. Despite the efficiency of this technique, sports nutrition professionals should proceed with care and attempt to cool the athletes when needed. Additionally, it is recommended to monitor an athlete’s body temperature and water loss and use this practice with the minimum applications and durations needed to achieve the desired loss in body mass.

###### 5.4.7.8. Mummy wraps – practical applications

- Though rarely used alone, the combination of other AWL modalities, such as training, hot baths, sauna, etc., prior to mummying allows the insulated heat to be maintained, thus extending the duration of sweating while minimizing heat stress.
- Duration of mummying is highly variable but due to the reduced heat stress compared to modalities such as a hot bath/sauna, longer durations of 15–30 min are common.
- Carefully monitor the athletes core temperature during any combination of hot baths, sauna, and mummying to prevent hyperthermia, 40°C and 104°F.

###### 5.4.6.9. Floating

Respiratory water loss is water that is expelled via pulmonary ventilation. Respiratory water is a byproduct of aerobic metabolism. Even in the absence of visible perspiration, approximately one-half of water turnover occurs through what is called insensible water loss, i.e. water lost from the lungs and skin [115]. Numerous factors can influence the amount of respiratory water losses. For instance, in temperate environments, these losses are approximately equal to the amount of water generated through aerobic metabolism: roughly 250–350 ml per day, increasing with respiratory rate as exercise intensity increases [168]. Large decreases in humidity (e.g. 80% to 20%) can significantly affect respiratory water losses, particularly during exercise where losses increase from 0.8 to 2.7 mL/min [169]. However, it appears that altitude doese not significantly alter respiratory water losses [188]. Oral exhalation compared to nasal exhalation also increases net respiratory water losses by up to 46% [189]. In practice, the ability to acutely manipulate this small amount of water relative to other forms of water manipulation strategies is minimal. Nonetheless, insensible water loss from respiratory water losses is often factored into acute water loss strategies and commonly coined the term “floating.” Increasing a combat athlete’s exposure to low humidity environments in the days leading to weigh-ins or particularly during sleep may provide an increase in water loss [152]. While the capacity for water loss through respiration is limited, particularly when floating at nighttime, combat athletes often require fluid losses in terms of ounces in the latest stages of acute water loss practices to successfully arrive at the required weight to compete.

#### 5.4.7. Fight week weigh-in protocols

The allotted recovery window between weigh-ins and competition dictates the magnitude of acute water loss. As noted, several combat sports disciplines require same-day weigh-ins with limited recovery windows. Olympic sports such as wrestling and Tae kwon do are such examples, as well as most of the governing bodies regulating jiu-jitsu competitions. Accordingly, rapid weight-loss strategies have diminished value to the athlete when the window of time available to replenish fluid and electrolyte losses is less than 4 h. Moreover, sports such as Brazilian jiu-jitsu and wrestling may have an athlete on the mat well within 2 h of their official weigh-in, depending on their weight class and how the competition is structured. As such, recovery windows that are short make it harder for meaningful rehydration and refueling to occur if excessive weight cuts are adopted. Therefore, careful planning to maintain an appropriate weight during the general preparation phases becomes of greater significance. Moreover, the longitudinal weight descent must be carefully designed to allow the athlete to decrease as much body mass as possible via changes in body composition and reductions in body fat. It is imperative to have the proper planning of total calories and macronutrient distributions coupled with training volumes. Based upon anecdotal observations from the authors working with thousands of combat athletes, below are recommended percentages of body mass loss that may be most appropriate for varying lengths of time between weigh-in and competition.

##### 5.4.7.1. Weigh-in timing

##### 5.4.7.1.1. Weigh-in < 4 h of competition

- Net 0–3% body mass loss from sweat losses starting in a euhydrated state.
- Nutritional Status: Glycogen stores should not be fully depleted prior to sweat loss, allowing for an additional 1–2% body mass loss from water bound in glycogen.
- Relevant Sports: Brazilian Jiu jitsu, wrestling, and most amateur combat sports weigh-ins.

##### 5.4.7.1.2. Weigh-in within 4–12 h of competition

- Net 2–4% body mass loss from sweat losses starting in a euhydrated state.
- Nutritional Status: Glycogen stores should not be fully depleted prior to sweat losses, allowing for an additional 1–2% body mass loss from bound water in glycogen.
- Relevant Sports: Combat sports with a morning weigh-in and an afternoon/evening competition (e.g. Amateur Muay Thai, Olympic Boxing),

##### 5.4.7.1.3. Weigh-in within 12–24 h of competition

- Net 3–5% body mass loss from sweat losses starting in a euhydrated state.
- Nutritional Status: Glycogen stores may be depleted prior to sweat losses given the adequate recovery window for glycogen restoration.
- Relevant Sports: Morning or evening weigh-in with competition the following day.

##### 5.4.7.1.4. Weigh-in within 24–36 h of competition

- Net 4–6% body mass loss from sweat losses starting in a euhydrated states.
- Nutritional Status: Glycogen stores may be depleted prior to sweat losses given the adequate recovery window for glycogen restoration.
- Relevant Sports: Professional MMA (UFC) or professional Muay Thai, Kickboxing, or Boxing.

## 6. Weigh-In Recovery

Prior to competition, combat athletes have their body mass verified by an official “weigh-in” to ensure that they meet the weight requirements for the competitive division. As noted, the time span between official weigh-ins varies among different combat sports, athletic commissions, promotions, and competition levels with recovery windows spanning 15 min up to 36 h. Considering this, combat athletes utilize an array of rapid weight loss and acute water loss strategies to manipulate their body mass, allowing them to compete in a lower weight class in hopes of attaining a physical size advantage [6]. When rapid weight loss and acute water loss strategies are conducted appropriately, sustainably, and safely, the physiological consequences and performance/health impairment can be reversed or mitigated by adopting effective post weigh-in nutrition and hydration recovery tactics. Key considerations for post weigh-in recovery include rehydration, glycogen restoration and gastrointestinal distress management [6].

### 6.1. Post weigh-in – rehydration

Olympic combat athletes commonly lose ~5% of their body mass in the week prior to weigh-ins [107], with professional MMA athletes in the UFC losing approximately 4.4% of their body mass 24 h before weigh-ins [177]. To reverse the physiological consequences of dehydration [190,191], a post weigh-in rehydration plan must consider the amount of weight lost from dehydration (i.e. sweat losses). General sports nutrition recommendations suggest replacing 125–150% of the fluid lost from sweat [192]. However, replacing such a large volume of fluid is attainable for recovery periods of 24–36 h, but it is often impractical for smaller recovery periods such as same-day weigh-ins.

To facilitate fluid assimilation, ingested fluids must first be consumed and emptied into the stomach, absorbed through the small intestines, and then enter the bloodstream. Several factors can influence gastric emptying, including energy density, volume of fluid bolus, fluid ingestion rate, osmolality, and pH [193]. Although gastric emptying rates can vary from person to person, they generally level off around 600 ml, with larger volumes (>1,000 ml) resulting in slower emptying [194]. Another important consideration is the reduced gastric emptying rate that occurs after intense exercise [195], hypohydration exceeding 3% of body mass [149], and the common practice of reducing food and fluid intake before weigh-ins. Taking these factors into account, for combat athletes undergoing acute weight loss from hypohydration greater than 3% of body mass, it is recommended to start with a conservative initial bolus of approximately 300–500 mL of fluid immediately after the official weigh-ins, followed by additional volumes (~240–350 mL) at regular intervals (every 30 min) to maintain gastric volume.

Post weigh-in rehydration should meet the electrolyte replacement needs of combat athletes. Electrolytes lost through thermoregulatory sweat losses are primarily sodium and chloride [192]; thus, replacing these key electrolytes allows for the optimal restoration of osmolality and fluid volume. Estimating total sodium losses from sweat can be challenging due to the large variations amongst individuals, however this typically ranges around ~20–80 mmol/dL [196]. This can be further complicated by the common practice among combat athletes of adopting a lower sodium diet prior to weigh-ins [6]. Despite this, sodium content correlates with fluid retention. Therefore, oral rehydration solutions (ORS) with a sodium range of 50–90 mmol/dL [192] should be utilized when hypohydration is >3% of body mass. For hypohydration <3% of body mass, commercial sports drinks (<30 mmol/dL sodium) result in adequate water retention. Rehydration can also be monitored in part by tracking urination rate and color as well as measuring the athlete’s weight as the rehydration protocol progresses.

#### 6.1.1. Post weigh-in carbohydrate

Carbohydrates are critical for the high-intensity nature of combat sports. Due to the practice of glycogen depletion prior to weigh-in to facilitate rapid weight loss, glycogen restoration following weigh-ins is desired [6]. Post weigh-in nutrition should include adequate carbohydrate content to at least fuel the athlete for the competition and maximize glycogen stores. Glycogen restoration must be carefully balanced and introduced at a tolerable rate to reduce gut discomfort while being at a low enough energy density to not hamper gastric emptying during the initial rehydration phase [194]. For this reason, an oral rehydration solution with a 2–3% carbohydrate (e.g. 15 g/500 ml) solution is more appropriate during rehydration for hypohydration >3% of body mass, whereas sports drinks with a ~ 5% carbohydrate (e.g. 25 g/500 ml) solution is well tolerated for hypohydration <3% of body mass.

With the initial oral rehydration solution and then solid foods, post weigh-in carbohydrate intake totaling 8–12 g/kg is appropriate for combat athletes that underwent significant glycogen depletion to facilitate rapid weight loss prior to weigh-ins. This range of carbohydrate ingestion is suitable for maximizing liver and skeletal muscle stores. However, if greater glycogen depletion has not occurred then aggressive carbohydrate intake is not necessary, and combat athletes may simply adopt a more moderate post weigh-in carbohydrate intake ranging 4–7 g/kg [6].

Strategies to meet carbohydrate needs post weigh-in include high glycemic index carbohydrates [197] and the use of carbohydrate rich fluids to simultaneously meet total fluid needs while facilitating glycogen restoration [6]. When ingesting carbohydrates at a rate of >60 g/h, incorporating food/fluids/supplements with a varied carbohydrate source (e.g. glucose and fructose) facilitates carbohydrate absorption via multiple gut transport mechanisms [198].

#### 6.1.2. Post weigh-in gastrointestinal distress management

Post weigh-in intake should prioritize optimizing nutritional status while avoiding GI distress. Of the many dietary modifications used to elicit rapid weight loss, a low residue diet has the most benign impact on performance and health [157]. In addition, fiber has a minimal contribution to energy needs and reduces gastric emptying/absorption rates and therefore the rapid reintroduction of fiber can cause discomfort [199] Fiber intake post weigh-in contraindicated for optimal post weigh-in recovery and should be limited. Protein and fat intake should be managed in the post weigh-in protocols as large boluses may also delay gastric emptying.

In addition, gastrointestinal distress following weigh-ins can be limited by avoiding trigger food/beverages during the acute rehydration phase (0–2 h following weigh-ins). Currently, no literature on specific foods that cause GI distress in combat athletes exists. However, certain foods are associated with gastrointestinal distress in both the general population and athletes [200,201]. Thus, the authors suggest avoiding or minimizing these foods:

- Coffee (independent of caffeine intake) relaxes lower esophageal sphincter.
- Citrus Fruits and Juices: lemon/lime, grapefruit and pineapple have high acid content.
- Tomatoes: tomato juice, tomato-based pasta sauces have high acid content.
- Carbonated Beverages: gaseous distention of the stomach and increases pressure on the lower esophageal sphincter causing reflux and bloating.
- Chocolate: contains compounds which relaxes the lower esophageal sphincter
- Peppermint, Garlic, Onion: relaxes the lower esophageal sphincter.
- Fatty, Spicy, Fried Food: delay gastric emptying and may exacerbate reflux symptoms, bloating and indigestion.

## 7. Fight day strategies

Fight day-specific nutrition strategies are targeted for athletes with a 24–36-h recovery window, as shorter recovery windows (<12 hr) are simply an extension of the weigh-in recovery/fueling strategies discussed earlier. In theory, athletes with 24+ hour recovery window adopt most (if not all) rapid weight loss and acute water loss strategies, including carbohydrate depletion, sodium reduction, and low residue diets in addition to sweat losses. At the same time, the long recovery window presents sufficient time for rehydration and refueling for peak performance. Fight day specific strategies can be summarized as providing athletes the refueling needs in a form, dosage and ingestion rate that minimizes gut disturbances yet meets and/or respects an athlete’s habits, traditions, and performance needs.

Fight day macronutrient intake should consist primarily of carbohydrates, as high-intensity sports (>70% VO2max) rely extensity upon carbohydrate as a fuel source [97]. Due to the range in competition times and variation in individual athlete sleep/recovery needs, a simple yet effective calculation for estimating fight day carbohydrate needs is 1–4 g/kg of body mass [97]. Carbohydrates with a high glycemic index and easily digestible starchy carbohydrates that an athlete habitually ingested throughout the preparation cycle are recommended. This may include (but not limited to): starchy tubers (potatoes/sweet potato), rice and flour-based products (sourdough/pancakes/pastas). Anxiety associated delays in-gastric emptying 2–3 h prior to competition may limit meal bolus tolerance [202]. Strategies to meet carbohydrate needs can include liquid sources of carbohydrates, e.g. sports drinks at 6–8% carbohydrate in addition to low volume carbohydrate snacks (bars, pretzels, applesauce, dates, etc.).

- *g. An athlete weighing 70 kg that wakes up at 08:00 for a fight time of 14:00 has 6 h to employ fight day nutrition strategies. Given the 6-h window, this athlete should ingest between 210-280 g of carbohydrates.*
- *g. An athlete weighing 70 kg that wakes up at 10:00 for a fight time of 22:00 has 12 h to employ fight day nutrition strategies. Given the 12-h window, this athlete should ingest between 420-560 g of carbohydrates.*

Protein intake can promote glycogen repletion, minimize muscle damage and promote a positive nitrogen balance [97]. In addition, protein co-ingested with carbohydrates before a training session/competition can promote glycogen repletion and prevent reactive or rebound hypoglycemia [203]. As for a dosing strategy, a modest intake pre-competition split into 20–30 g meal servings will meet protein needs without displacing carbohydrate intake [97].

- *g. An athlete weighing 70 kg that wakes up at 08:00 for a fight time of 14:00 has 6 h to employ fight day nutrition strategies. Given the 6-h window, this athlete should ingest between 50-70 g of protein in 20-30 g/meal boluses.*
- *g. An athlete weighing 70 kg that wakes up at 10:00 for a fight time of 22:00 has 12 h to employ fight day nutrition strategies. Given the 12-h window, this athlete should ingest between 100-140 g of protein in 20-30 g/meal boluses.*

Although calorically dense, fats are less of a priority due to the high-intensity nature of combat sports. Rather than adding high fat sources to meals (e.g. cream sauces) or ingesting fat-rich snacks (e.g. tree nuts), the fat calories should simply be a byproduct of the food source itself (e.g. eggs). It should also be noted that due to reduced gastric emptying effect of fats [204] compounded by pre-fight stress and anxiety [205], fat intake should be avoided 3 h prior to competition.

Respecting athlete traditions and habits is of equal importance to performance nutrition and gut tolerance-based fight day strategies. The sport nutrition professional should maintain cultural competence when recommending fight day dietary strategies especially for athletes of diverse cultures in a way that is effective and appropriate. Fight day dietary strategies should consist of food and beverage options that are familiar and habitually consumed through the athlete’s competition preparation. In addition, rigid and uncompromising fight day dietary strategy may fail to accommodate for the chaotic nature of combat sports fight day logistics which may have frequent interruptions due to increased sleep/recovery needs, preference for fight day “shakeouts” or training sessions, changes to bout times/fight card logistics and unaccounted for anxiety-induced food intolerance. A flexible approach utilizing an array of food and beverage sources that are easily available, familiar and performance nutrition oriented is the best approach for meeting an athlete’s fight day nutrition needs.

### 7.1. Refueling and rehydration – practical applications

The following practical applications are based on rehydration strategies used in clinical settings and current practices used by combat athletes [116,150,166].

Acute Rehydration (1–2 h post weigh-in)

- Utilize oral rehydration solution (1 to 1.5 liters per hour)
- Utilize fast-acting carbohydrates with low osmolarity at a tolerable rate of ≤60 grams per hour.
- Re-introduce easily digestible starchy carbohydrates as tolerated (i.e. saltines, bagels, rice cracker, etc.)

Non-acute Rehydration (3–6 h post weigh-ins)

- Fluid must contain electrolytes: primarily sodium, potentially also potassium and chloride.
- Utilize simple and easily digestible starchy carbohydrates as tolerated (i.e. white rice, potatoes, sweet potatoes, pasta, etc.)

Muscle Glycogen Restoration Phase (6+ hours Post Weigh-in)

- Prioritize carbohydrate intake, ingesting only low to moderate protein.
- Avoid high-fat foods, as these will delay gastric emptying. Eat small frequent meals, rather than a single large bolus.

Weight Regain

- As the combat athlete refuels and rehydrates, monitor urination, urine color, and increases in body mass.
- At least a 10% increase in body mass post weigh-in may be optimal for fight performance and reducing health consequences.

## 8. Final summary and conclusions

Combat sports encompass a range of tactics and skills that involve direct one-on-one physical contact with the ultimate objective of overpowering, striking, or submitting one’s opponent. These disciplines attract athletes at various levels, ranging from recreational and amateur competitions to professional and Olympic events. One distinguishing feature of combat sports is the implementation of weight divisions, which serve to categorize athletes based on their body mass. This ensures that competitors face opponents who are of comparable size and weight, promoting fairness and safety in the sport. To compete in their desired weight class and potentially gain a competitive edge, athletes resort to weight-loss practices that can be both sustained/longitudinal and rapid. Accordingly, it is crucial to consider the nutritional and weight cut strategies of training and competition in combat sports since they can significantly impact an athlete’s performance and overall well-being. Distinct phases of training, such as the general preparation/off camp, fight camp, and fight week, all require specific strategies to optimize performance and support the athlete’s health. When designing nutritional and weight cut strategies for combat athletes, factors such as body mass, body composition, type of combat sport, duration of the fight camp, and the time between weigh-in and the actual fight must be considered.

The following 16 points constitute the Position Statement of the Society. The Research Committee of the Society has approved them:

1. Combat sports have differing weight categories, official weigh-in times, and competition frequencies, influencing the nutritional and weight cut strategies for training and competition.
2. As the duration of a combat match increases, >4 minutes, contribution of the aerobic system can rise to >70%, yet anaerobic alactic pathways and anaerobic glycolytic pathways support high-output bursts.
3. During the off camp/general preparation phase, athletes should maintain a weight ranging 12% to 15% above the weight division requirement.
4. Supplements including creatine, beta-alanine, beta-hydroxy-beta-methylbutyrate, and caffeine have been shown to enhance performance and/or recovery during preparation phases, competition, and post-competition.
5. During fight camp, strategic decreases in calorie intake are necessary for an efficient longitudinal weight descent. Individual caloric needs can be determined using indirect calorimetry or validated equations such as Mifflin St. Jeor or Cunningham.
6. Protein should be prioritized during longitudinal weight descents to preserve lean body mass, and the timely delivery of carbohydrates supports training demands. Macronutrients should not drop below the following: carbohydrates 3.0–4.0 g/kg, protein 1.2–2.0 g/kg, and fat 0.5 to 1.0 g/kg/day.
7. Suitable losses in body mass range from 6.7% at 72 h, 5.7% at 48 h, and 4.4% at 24 h, prior to weigh-in.
8. Sodium restriction and water loading are effective for inducing polyuria and acute water loss.
9. During fight week, water-bound glycogen stores can be depleted through exercise and carbohydrate restriction, facilitating a 1 to 2% loss in body mass, with equivalent losses from a low fiber intake, <10 g/day for 4 days.
10. During fight week, acute water loss strategies, including sauna, hot water immersion, and mummy wraps, can be used effectively with appropriate supervision (optimally ~2–4% of body mass within 24 h of weigh-in).
11. Post-weigh-in, rapid weight gain strategies are utilized to recover lost body fluid/mass before competition with the intent of gaining a competitive advantage.
12. Oral rehydration solutions (1 to 1.5 liters/h) combined with a sodium range of 50-90 mmol/dL should take precedence immediately post-weigh-in.
13. Fast-acting carbohydrates at a tolerable rate of ≤ 60 grams/h should follow oral rehydration solutions. Post weigh-in intake of fiber should be limited to avoid gastrointestinal distress.
14. Post-weigh-in carbohydrate intake at 8–12 g/kg may be appropriate for combat athletes that undertook significant glycogen depletion strategies during fight week. 4-7 g/kg may be suitable for modest carbohydrate restriction.
15. Post weigh-in, rehydration/refueling protocols should aim to regain ≥10% of body mass to mitigate declines in performance and the negative effects of rapid weight loss.
16. The long-term effects of frequent weight cuts on health and performance are unknown, necessitating further research.
